# Development of synthetic modulator enabling long-term propagation and neurogenesis of human embryonic stem cell-derived neural progenitor cells

**DOI:** 10.1186/s40659-023-00471-0

**Published:** 2023-11-11

**Authors:** Ceheng Liao, Ying Guan, Jihui Zheng, Xue Wang, Meixia Wang, Zhouhai Zhu, Qiyuan Peng, Hong-Hui Wang, Meng Li

**Affiliations:** 1 College of Biology, Hunan University, 27 Tianma Road, Yuelu District, Changsha, 410082 Hunan China; 2 Joint Institute of Tobacco and Health, 367 Hongjin Road, Wuhua District, Kunming, 650202 Yunnan China

## Abstract

Neural progenitor cells (NPCs) are essential for in vitro drug screening and cell-based therapies for brain-related disorders, necessitating well-defined and reproducible culture systems. Current strategies employing protein growth factors pose challenges in terms of both reproducibility and cost. In this study, we developed a novel DNA-based modulator to regulate FGFR signaling in NPCs, thereby facilitating the long-term maintenance of stemness and promoting neurogenesis. This DNA-based FGFR-agonist effectively stimulated FGFR1 phosphorylation and activated the downstream ERK signaling pathway in human embryonic stem cell (HESC)-derived NPCs. We replaced the basic fibroblast growth factor (bFGF) in the culture medium with our DNA-based FGFR-agonist to artificially modulate FGFR signaling in NPCs. Utilizing a combination of cell experiments and bioinformatics analyses, we showed that our FGFR-agonist could enhance NPC proliferation, direct migration, and promote neurosphere formation, thus mimicking the functions of bFGF. Notably, transcriptomic analysis indicated that the FGFR-agonist could specifically influence the transcriptional program associated with stemness while maintaining the neuronal differentiation program, closely resembling the effects of bFGF. Furthermore, our culture conditions allowed for the successful propagation of NPCs through over 50 passages while retaining their ability to efficiently differentiate into neurons. Collectively, our approach offers a highly effective method for expanding NPCs, thereby providing new avenues for disease-in-dish research and drug screening aimed at combating neural degeneration.

**Supplementary Information:**

The online version contains supplementary material available at 10.1186/s40659-023-00471-0.

## Introduction

Neurological disorders, such as Alzheimer’s disease, Parkinson’s disease, and traumatic brain injuries, present significant global health challenges, affecting millions of individuals worldwide and leading to substantial impairment in the quality of life for patients and their families [1–3]. Understanding the underlying pathology of these disorders and developing effective therapies necessitate a comprehensive understanding of neural cell behavior and the ability to manipulate and study neural progenitor cells (NPCs) [4, 5]. NPCs are a critical cell population in the field of neuroscience research and regenerative medicine due to their unique properties, including self-renewal and the ability to differentiate into various cell types within the nervous system, such as neurons, astrocytes, and oligodendrocytes [6, 7]. This remarkable versatility makes NPCs invaluable for a wide range of applications, such as studying disease mechanisms, conducting drug screenings, and developing cell-based therapies to treat or even reverse the effects of neurological disorders [8–10].

The practical application of NPCs in disease modeling and regenerative medicine heavily depends on the ability to culture and expand these cells on a large scale. Establishing reliable and scalable culture systems for NPCs is crucial to ensure a sufficient supply of cells for experimentation and therapeutic purposes. Large-scale culturing of NPCs offers the potential to generate high-quality cellular models that accurately recapitulate disease-associated features, providing valuable insights into disease mechanisms and enabling the development of novel therapeutic interventions [11, 12]. The ability to culture and expand NPCs on a large scale allows scientists to perform extensive drug screenings, testing the efficacy and safety of potential treatments in a controlled and reproducible manner [13, 14]. Establishing reliable and scalable culture systems for NPCs can unlock the full potential of these versatile cells, thus paving the path for groundbreaking discoveries and therapeutic advancements in neuroscience and regenerative medicine [15].

The expansion and maintenance of NPCs in neuroscience research and regenerative medicine face significant challenges, such as establishing a well-defined, reproducible culture system and managing high production costs associated with traditional methods [16, 17]. Conventional NPC culture systems commonly employ serum-free media, enriched with specific protein growth factors such as fibroblast growth factor (FGF), epidermal growth factor (EGF), and brain-derived neurotrophic factor (BDNF) [18, 19]. These growth factors are pivotal in maintaining the self-renewal and differentiation capacities of NPCs, and the specific isoforms of these receptors can vary depending on the origin of the NPCs in vivo. However, these methods present challenges, such as batch-to-batch variability in serum-containing media [20], potential immunogenicity [21], and elevated production costs [22]. To address these challenges, researchers are exploring alternative approaches, such as chemically defined media and small molecules as substitutes for growth factors, aiming to establish a more consistent and cost-effective culture system while preserving stem cell properties and differentiation potential of NPCs [23, 24]. These promising strategies, such as the development of chemically defined media and synthetic substitutes for serum or growth factors, offer improved consistency, reproducibility, and cost-effectiveness by offering greater control over the culture environment, eliminating batch-to-batch variability, and reducing the risk of immunogenic responses [25, 26]. Therefore, developing chemically defined media would facilitate the establishment of NPC cultures, ultimately improving reproducibility and increasing versatility for exploring their applications in disease modeling and therapeutics.

**Scheme 1:**
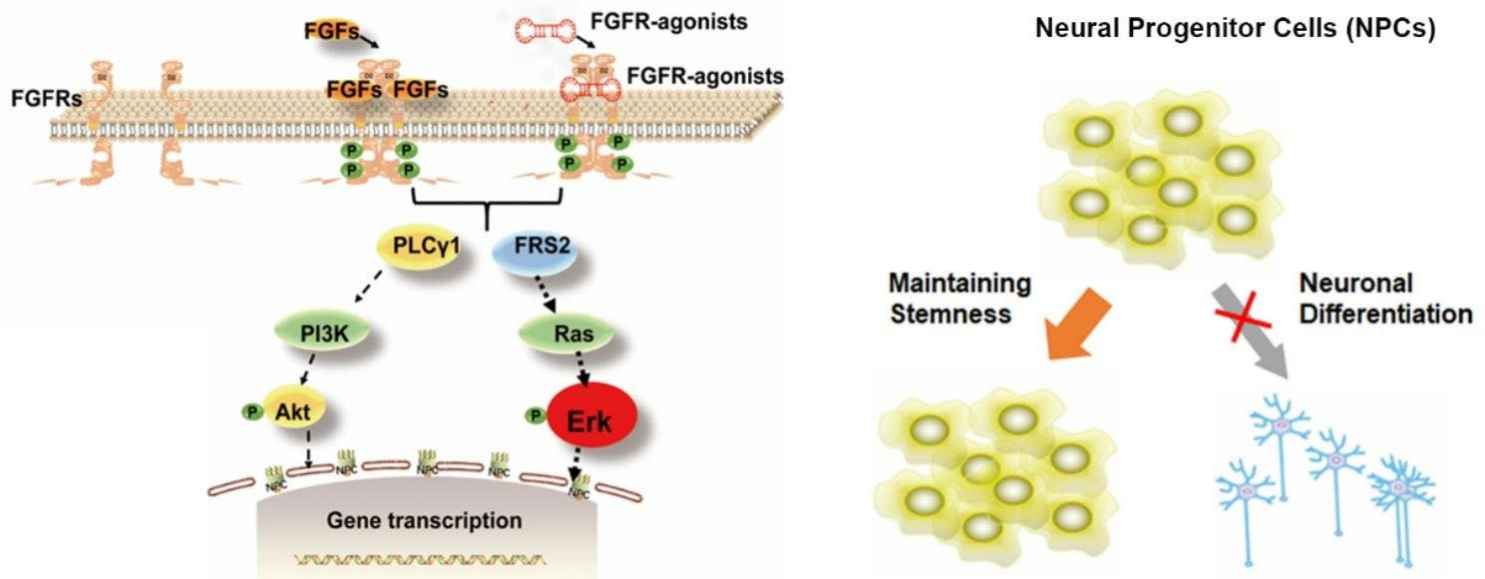
DNA-based FGFR-agonist for NPCs maintenance

In this study, we developed a novel approach that utilizes a DNA-based modulator to maintain the stemness of NPCs and facilitate the expansion of NPCs (Scheme 1). By replacing protein-based native growth factors with DNA-based modulators, we aimed to overcome the limitations of current NPC culture systems, such as experimental variability and high production costs [27, 28]. We investigated the potential of an artificial DNA-based modulator as a viable alternative to bFGF to maintain and expand human-derived NPCs and decouple the default neuronal differentiation program. To achieve this, we conducted a comprehensive evaluation of DNA modulator-mediated FGFR signaling and transcriptome analysis and assessed the maintenance of stemness in NPCs. By utilizing the FGFR-agonist, we established a chemically defined culture system that effectively preserves the stem cell characteristics of NPCs. We expect this novel approach has the potential to revolutionize the field of neuroscience research and regenerative medicine by offering a more consistent, scalable, and cost-effective method for the large-scale production of NPCs.

## Results

### Design and characterization of DNA-based FGFR-agonist

**Fig. 1:**
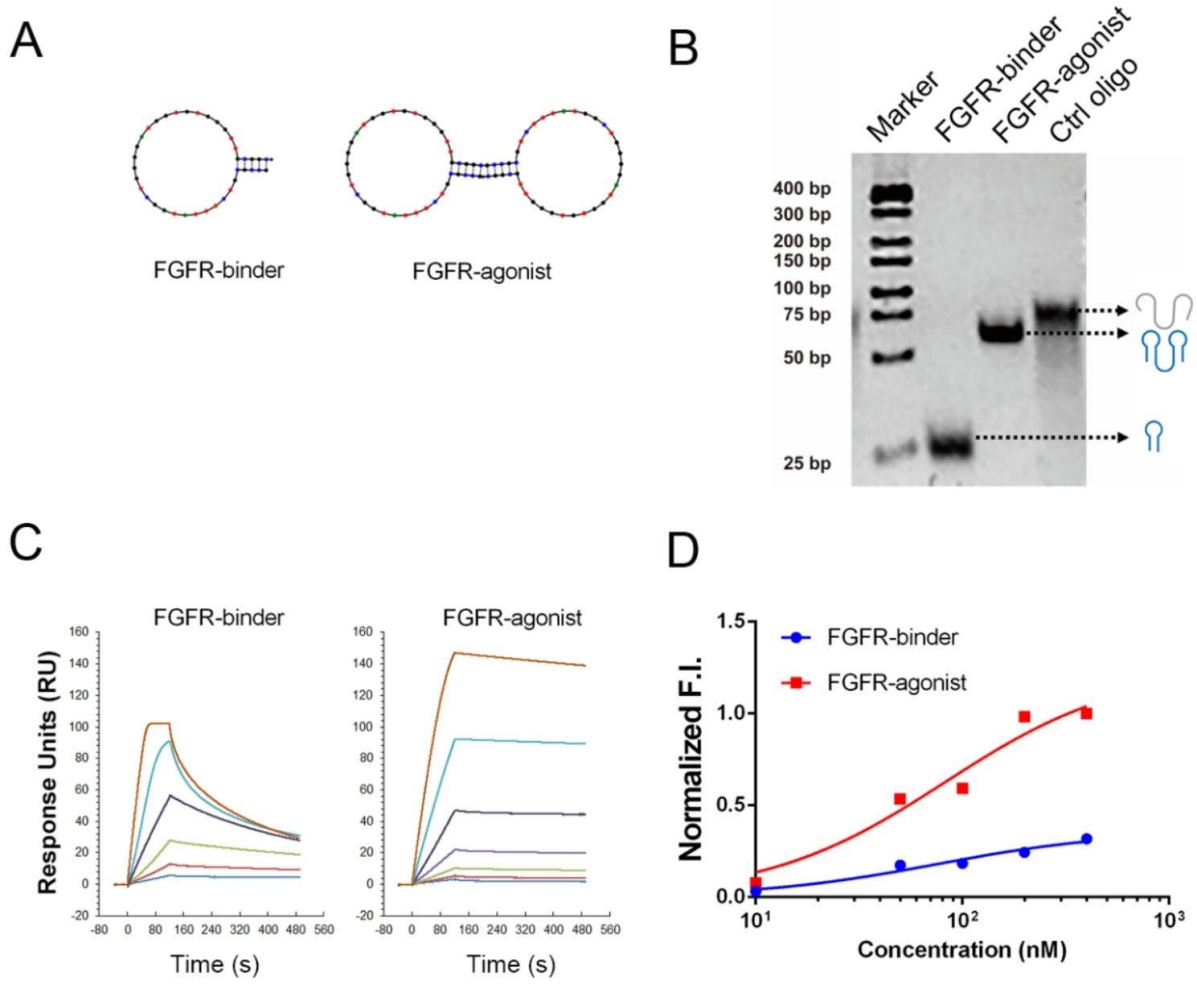
**Design and characterization of FGFR-agonist.** (**A**) The secondary structures of the FGFR-binder and FGFR-agonist determined using Mfold software are presented, indicating the formation of a stem-loop structure. (**B**) The secondary structure of DNA-based FGFR-agonist is verified using native polyacrylamide gel electrophoresis (PAGE). This panel also includes the determined molecular weights of the single-stranded FGFR-binder, FGFR1-agonist, and a control oligonucleotide (Ctrl oligo). (**C**) SPR sensorgrams showing the real-time binding kinetics of FGFR-agonist aptamer to immobilized FGFR1 extracellular domain at various concentrations. The sensorgrams are color-coded based on the concentrations of the FGFR-binder (1, 2, 4, 8, 16 and 32 nM) and FGFR-agonist (0.16, 0.31, 0.625, 1.25, 2.5, 5 and 10 nM), respectively. (**D**) The relative binding performance of FGFR-binder and FGFR-agonist to the NIH3T3 cells was determined by flow cytometry (refer to **Fig. **S1 for additional details)

The essential role of FGF-FGFR1 signaling in NPCs is well-documented [29]. Previous research has also demonstrated the development of a bivalent FGFR1 agonist to maintain and support the pluripotency of human embryonic stem cells (HESCs) [30]. Similar strategies for developing DNA-based agonists have been previously employed to mimic HGF and VEGF [31, 32]. However, the effort to use DNA-based receptor agonists for NPC stemness maintenance remains unexplored. In this study, we intended to utilize a DNA-based synthetic agonist to mimic the function of bFGF, allowing for controlled receptor dimerization and effective activation of FGFR signaling in NPCs. Similar to the previous concept, we designed a bivalent DNA architecture containing two FGFR1-binding domains that could facilitate FGFR1 dimerization. We employed a previously characterized monomeric FGFR-binding aptamer, a 38-mer stem-loop oligonucleotide, as our FGFR1 binder (Fig. 1A, **Table ****S1**). To induce ligand-mediated receptor dimerization, we conjugated two monomeric FGFR1 binders to create a bivalent ligand capable of assembling two cell surface FGFR1 molecules, acting as an FGFR1 agonist. Structural predictions indicated that the bivalent configuration did not adversely affect the secondary structure of each FGFR1-binding domain (Fig. 1A). Using 8% Native PAGE gel electrophoresis, we observed that the molecular weight of a single-stranded FGFR binder was approximately 25 nucleotides (nt). In contrast, the molecular weights of both the bivalent FGFR1 agonist and its control oligonucleotide were around 72 nt, likely due to their respective secondary structures (Fig. 1B). Notably, the control oligonucleotide (Ctrl-oligo), which has same number of nucleotides with a scrambled sequence compared to the FGFR1 agonist, displayed a higher molecular weight during electrophoretic analysis. This observation may be attributed to the G-quadruplex (G4) structure possessed by the FGFR1 agonist, which leads to a more compact conformation in native PAGE assays [30]. In contrast, the scrambled sequence in the control group disrupts this ordered structure, resulting in a more linear spatial configuration. Consequently, the electrophoretic migration rate slows down, manifesting as a higher molecular weight. This data also suggests that the compromised structure of the Ctrl-oligo could adversely affect its ability to effectively interact with FGFR1.

To elucidate the molecular binding properties of the monomeric FGFR-binder and bivalent FGFR1-agonist with the extracellular domain of FGFR1, we employed Surface Plasmon Resonance (SPR) analysis using the Biacore method. Our results revealed that both FGFR-binder and FGFR1-agonist exhibited high binding activity against FGFR1 protein (Fig. 1C). However, there are distinct differences in the binding kinetics and affinities between the two constructs (Fig. 1C **and Table S2**). The FGFR-binder displayed a dissociation constant (KD) of 2.5 nM, while the FGFR1-agonist demonstrated a substantially improved affinity, with a KD value reaching 46.1 pM. The binding kinetics analysis revealed that the FGFR-binder had a fast association rate (K_a_) of 2.20 × 10^10^ Ms^− 1^ and a dissociation rate (K_d_) of 54.9 s^− 1^, indicating a rapid off-rate (**Table S2**). In contrast, the FGFR-agonist had a Ka of 4.51 × 10^6^ Ms^− 1^ and a K_d_ of 2.08 × 10^− 4^ s^− 1^. The dissociation speed of the FGFR1-agonist was significantly slower than that of the FGFR-binder, implying a more stable binding interaction between FGFR-agonist and FGFR1 protein, probably due to the multivalent interactions facilitated by the bivalent FGFR-agonist.

We next examined the binding activity of FGFR-agonist to the receptors on living cells using flow cytometry. We employed an FGFR1-positive NIH3T3 cell line, a murine fibroblast cell line. The results demonstrated that the FGFR1-binder exhibited a high affinity for FGFR1 with a KD of 39 nM (Fig. 1D and S1). Notably, the bivalent FGFR1-agonist could affect the cell-binding affinity compared with the monomeric FGFR-binder while significantly increasing the binding fraction, consistent with the previous finding in vitro SPR experiment. To explore the functionality of FGFR-agonist on the FGFR activation, the serum-starved NIH3T3 were stimulated with the FGFR-agonist and bFGF for 10 min, and the phosphorylation of FGFR1 (Tyr653/654) was evaluated using an enzyme-linked immunosorbent assay (ELISA). The results showed that the FGFR-agonist significantly promoted the phosphorylation level of FGFR1 compared with the controls treated with the monomeric FGFR-binder (**Fig.** S2). Moreover, the stability of the FGFR-agonist under physiological conditions would be highly important for its robust effect for in vivo application. To characterize this feature, we performed a serum stability assay by incubating the FGFR-agonist with 10% FBS at 37 °C. After incubation for different durations, we analyzed all samples using 8% Native PAGE gel electrophoresis. The results showed that the FGFR-agonist remained stable after 16 h in 10% FBS, indicating its suitability for subsequent experiments (**Fig.** S3).

### FGFR-agonist promotes FGFR signaling in NPCs

Haven successfully constructed a serum-stable DNA-based FGFR-agonist with high binding affinity for FGFR1, we next explored the potential FGFR-agonist in modulating the FGFR signaling pathway of NPCs. We obtained HESC-derived NPCs using a previously described pipeline with different combinations of culturing medium (**Fig.** S4). Immunofluorescence analysis confirmed the positive staining of these cells with Nestin and Pax 6, both of which are NPC markers (**Fig. **S5). Upon neuronal differentiation without bFGF, the NPCs were able to differentiate into MAP2-positive neurons (**Fig. **S6).

**Fig. 2:**
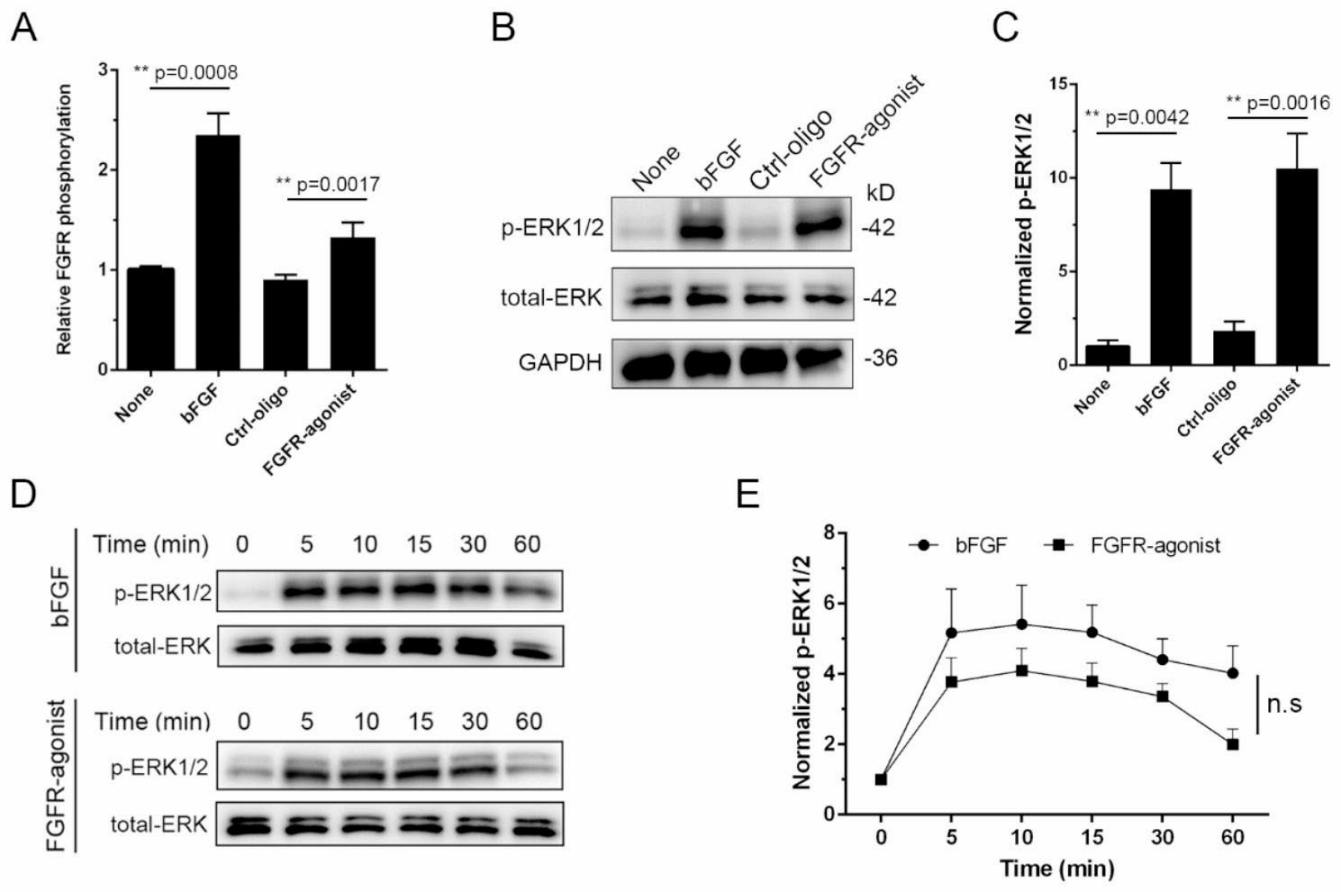
**FGFR-agonist promotes FGFR signaling of NPCs.** (**A**) ELISA-based quantification of FGFR1 phosphorylation in NPCs. FGFR1 (Tyr653/654) phosphorylation levels were measured in serum-starved NPCs after 10-minute exposure to either bFGF (20 ng/mL) or FGFR-agonist (40 nM). Data are represented as mean ± SD (n = 3); **p < 0.01(unpaired two-tailed Student’s t-test). (**B**) ERK1/2 phosphorylation profile via Western Blot. Serum-starved NPCs were treated with either bFGF (20 ng/mL) or FGFR-agonist (40 nM) for 10 min. Phosphorylation of ERK1/2 (Thr202/Tyr204) was then examined by Western blot, with GAPDH serving as an internal control. (**C**) Quantification of relative ERK1/2 phosphorylation to total ERK. The phosphorylation levels of ERK1/2 were quantified and normalized to the control group without any treatment. Data are expressed as means ± SD (n = 3); **p < 0.01(unpaired two-tailed Student’s t-test). (**D**) Time-dependent Activation of ERK1/2. Serum-starved NPCs were exposed to either bFGF (20 ng/mL) or FGFR-agonist (40 nM) over varying time intervals (5, 10, 15, 30, 60 min). The phosphorylation of ERK1/2 (Thr202/Tyr204) was assessed via Western blot. (**E**) Quantification of the time-dependent data from panel D, illustrating the kinetics of ERK1/2 phosphorylation in NPCs when stimulated by either bFGF or FGFR-agonist. Data are presented as mean ± SD (n = 3); no significance (n.s.) was found, as determined by one-way ANOVA

To evaluate the effectiveness of the FGFR-agonist in activating FGFR1, we employed an ELISA to quantify the phosphorylation levels of FGFR1 (Tyr653/654) in NPCs. Following a 24-hour period of serum starvation to synchronize cellular responsiveness, NPCs were exposed for 10 min to either bFGF or our custom-synthesized FGFR-agonist. Notably, the FGFR-agonist treatment resulted in a significant 1.4-fold increase in FGFR1 phosphorylation compared to the control group treated with Ctrl-oligo (Fig. 2A). Although the phosphorylation levels elicited by bFGF were higher (2.4-fold), our findings indicate that FGFR-agonist effectively mimics the activation properties of the natural ligand, bFGF. In parallel, we explored the activation of downstream signaling pathways, focusing on ERK1/2 phosphorylation at residues Thr202/Tyr204. Western blot analyses confirmed a pronounced increase in ERK1/2 phosphorylation levels for NPCs treated with either FGFR-agonist or bFGF when compared to both untreated controls and Ctrl-oligo treated cells (Fig. 2B and S7). Quantitatively, the ERK1/2 phosphorylation levels were amplified 10.5-fold in the FGFR-agonist group relative to the Ctrl-oligo group, whereas the bFGF-treated group showed a 9.4-fold increase relative to the control group (Fig. 2C).

To understand the temporal characteristics of FGFR-agonist-mediated signaling, we treated serum-starved NPCs with either bFGF or FGFR-agonist across multiple time intervals. The kinetics of ERK1/2 phosphorylation in response to FGFR-agonist were found to parallel those induced by bFGF (Fig. 2D). This corroborates that FGFR-agonist is comparably effective as bFGF in sustaining FGFR/ERK signaling over time (Fig. 2E). We also validated these findings across different cell lines by employing ATDC5 cells, a murine chondrocyte line that is responsive to bFGF stimulation [33]. We observed similar trends in FGFR1 and ERK1/2 phosphorylation, confirming the broad efficacy of the FGFR-agonist. Interestingly, bFGF elicited more sustained ERK phosphorylation in ATDC5 cells than FGFR-agonist, which differs from that shown in NPCs (**Fig. **S9). This variation could be attributed to differences in FGFR1 endocytosis across different cell types, which may consequently alter the kinetics of downstream signaling.

Collectively, our findings establish the role of the FGFR-agonist as an effective modulator of FGFR/ERK signaling, underscoring its ability to precisely modulate cellular signaling pathways and illuminate its potential as a potent tool for manipulating cellular behavior in NPCs and perhaps other FGFR-positive cell types.

### FGFR-agonist recapitulates the function of bFGF to modulate NPCs

**Fig. 3:**
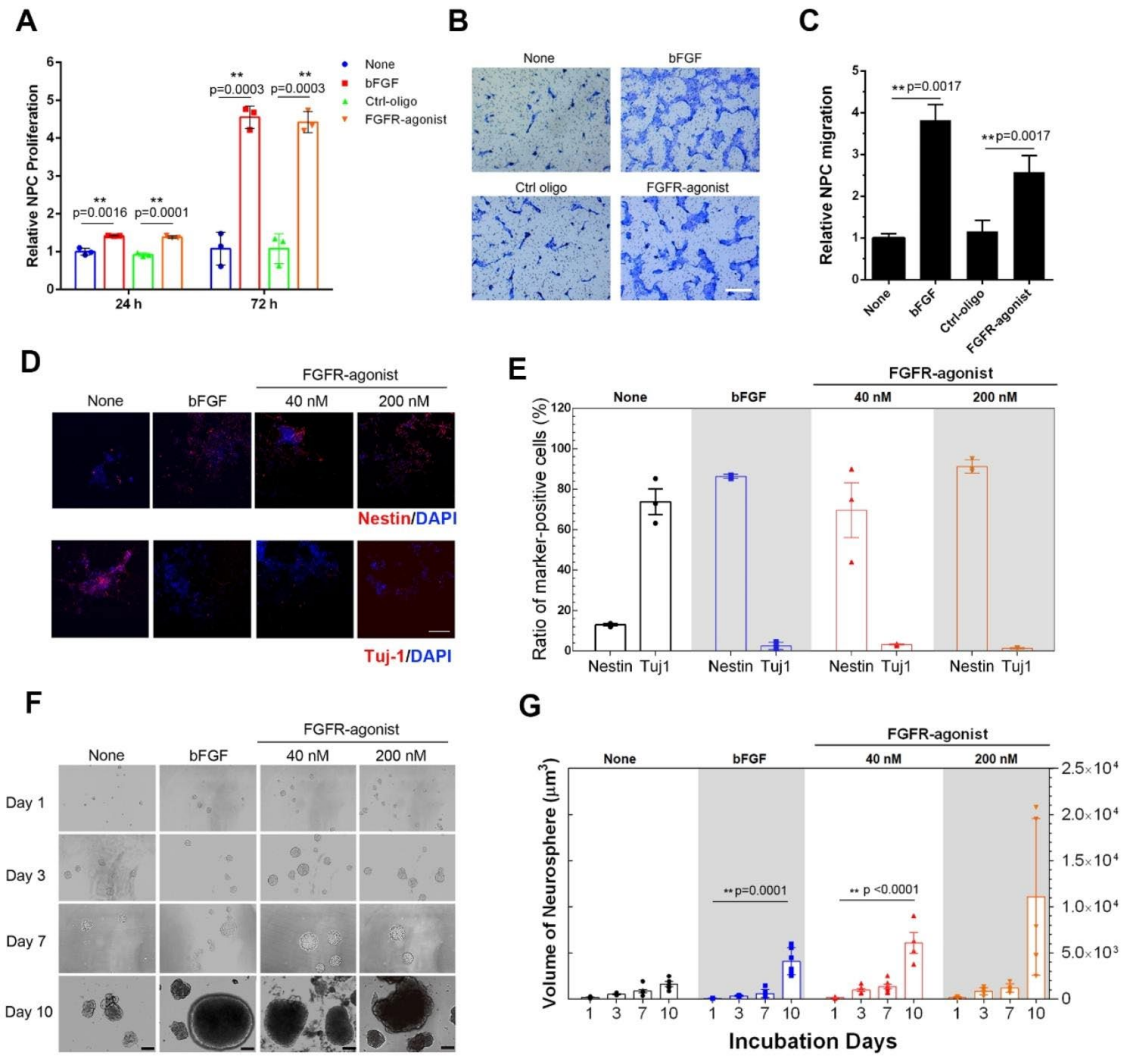
**FGFR-agonist regulates cellular behaviors of NPCs.** (**A**) The proliferation of NPCs was evaluated at 24-hour and 72-hour time points following treatments with bFGF or FGFR-agonist, using a CCK8 kit. (**B**) Serum-starved NPCs were placed in the upper chamber, while bFGF(20 ng/mL) and FGFR-agonist (40 nM) were included in the lower chamber for 24 h and stained, followed visualization under light microscopy. Scale bar = 100 μm. (**C**) The data from the Transwell assay are quantified to assess the impact of different treatments on NPC migration. Data are presented as mean ± SD (n = 6). (**D**) Representative images showing the undifferentiated NPCs (Nestin-positive) and differentiated neurons (Tuj-1 positive) NPCs treated with bFGF, FGFR-agonist and Ctrl-oligo for 7 days and subjected to immunofluorescence staining. Scale bar = 100 μm. (**E**) The ratios of Nestin-positive cells and Tuj-1-positive cells are quantified against total cells (DAPI-positive). Data are presented as mean ± SD (n = 3), with **p < 0.001, as determined by unpaired t-tests. (**F**) Representative images of neurospheres generated by NPCs cultured in suspension for 10 days in the presence or absence of bFGF (20 ng/mL), FGFR-agonist (40 nM), or FGFR-agonist (200 nM). The scale bar = 100 μm. (G) Quantification of the changes in neurosphere volume over a 10-day culture period under various treatment conditions. Data are displayed as mean ± SD (n = 3), with **p < 0.0001, as determined by one-way ANOVA

Building on our previous findings concerning the role of the FGFR/ERK signaling pathway in regulating essential cellular functions, we further examined the effect of FGFR-agonist on the proliferation of NPCs. Employing the CCK8 assay, we found that FGFR-agonist markedly promoted NPC proliferation during both 24-hour and 72-hour treatment periods, achieving levels similar to those observed with bFGF treatment (Fig. 3A). Remarkably, a 72-hour treatment with FGFR-agonist led to a 4.4-fold increase in cell proliferation when compared to the control group treated with Ctrl-oligo. This result closely paralleled the 4.6-fold increase seen in the bFGF-treated group. We further assessed the directional migratory capabilities of NPCs upon stimulation with bFGF and FGFR-agonist employing a Transwell migration assay. The results demonstrated that FGFR-agonist significantly elevated the number of migrated NPCs compared to the control group treated with Ctrl-oligo (Fig. 3B and S10). Quantitative analysis revealed that FGFR-agonist increased the cell migration rate by 2.8-fold relative to the Ctrl-oligo group. In contrast, recombinant protein bFGF exhibited a stronger ability to induce directional migration, achieving a 5.6-fold increase compared to the control group (Fig. 3C). Given the previously observed similarity in the proliferative effects between FGFR-agonist and bFGF, these data suggest that the differential migration responses might be due to biased signaling activation, warranting further investigation into the underlying mechanisms. In addition, the function of FGFR-agonist was extended to the ATDC5 cells, where FGFR-agonist similarly promoted proliferation and enhanced wound closure rates, comparable to the effects of bFGF (**Fig. S9**). Therefore, our findings underscore the potential of FGFR-agonist as an effective alternative or adjunct to bFGF for stimulating both NPC proliferation and migration.

Given the critical role of FGFR signaling in maintaining the stemness of NPCs, as indicated in previous research [18], we further explored the ability of FGFR-agonist to sustain the undifferentiated state of NPC. For this purpose, NPCs were treated with bFGF, FGFR-agonist, or Ctrl-oligo for 7 days. Immunofluorescence assays were performed to assess the expression levels of the stem cell marker Nestin and the neuronal differentiation marker Tuj-1. Our observations revealed that NPCs treated with either FGFR-agonist or bFGF retained a progenitor-like morphology and exhibited robust Nestin expression while showing minimal Tuj-1 levels (Fig. 3D **and Fig.** S11). In contrast, untreated cells or those treated with Ctrl-oligo displayed an elongated, neuron-like morphology and included some apoptotic cells, suggesting spontaneous differentiation (Fig. 3D). Quantitative analysis further substantiated these findings. The treatment with 40 nM of FGFR-agonist led to approximately 70% of cells being Nestin-positive and only 3.2% being Tuj-1-positive neurons (Fig. 3E). Treatment with 200 nM of FGFR-agonist resulted in an even higher proportion of Nestin-positive NPCs (91%) with a low ratio of neurons expressing Tuj-1 (1.3%). For comparison, bFGF treatment yielded 86% Nestin-positive cells and 2.5% Tuj-1-positive neurons (Fig. 3E). Collectively, our data strongly suggest that FGFR-agonist, much like bFGF, is effective in maintaining the undifferentiated state of NPCs and limiting spontaneous neuronal differentiation.

We next sought to investigate its potential utility in facilitating neurosphere formation, an essential procedure for obtaining neural progenitor cells for therapeutic applications. To this end, we compared its effects with bFGF, a standard growth factor ubiquitously used in NPC cultures. NPCs were cultured in a suspension system for a 10-day period, and two concentrations of FGFR-agonist (40 nM and 200 nM) were evaluated alongside a standard dose of bFGF (20 ng/mL). We evaluated several key parameters, including the number, size, and morphology of the neurospheres, to gauge the effectiveness and quality of neurosphere formation. Our preliminary findings showed that FGFR-agonist had a favorable impact on both the size and quantity of the neurospheres, akin to the effects observed with bFGF (Fig. 3F). In the presence of FGFR-agonist, neurospheres displayed morphological integrity comparable to, or even better than, those maintained with bFGF throughout the 10-day culture period (Fig. 3F). Conversely, the untreated control group merely formed rudimentary cell clusters within the initial five days. Subsequent quantitative analyses revealed that the dimensions of the neurospheres in the FGFR-agonist-treated groups were on par with those in the bFGF-treated group (Fig. 3G). By day 3, a clear divergence was evident in the growth trajectories between the neurospheres treated with bFGF or FGFR-agonist (40 nM) and those in the untreated group. This distinction persisted until day 10 when neurospheres in the active treatment groups continued to expand while those in the control group began to disintegrate. Furthermore, our 7-day treatment study revealed stable maintenance of neurosphere circularity in both bFGF and FGFR-agonist treatment groups. Specifically, treated neurospheres exhibited significantly higher circularity levels than untreated cells or those treated with Ctrl-oligo (**Fig. S12**). Collectively, these results validate the utility of FGFR-agonist as an effective substitute or supplement for bFGF in the formation and maintenance of neurospheres, providing compelling evidence that FGFR-agonist can emulate the biological roles of bFGF in regulating NPC behavior and promoting neurosphere development.

### FGFR-agonist affects the transcriptome program of NPCs

**Fig. 4:**
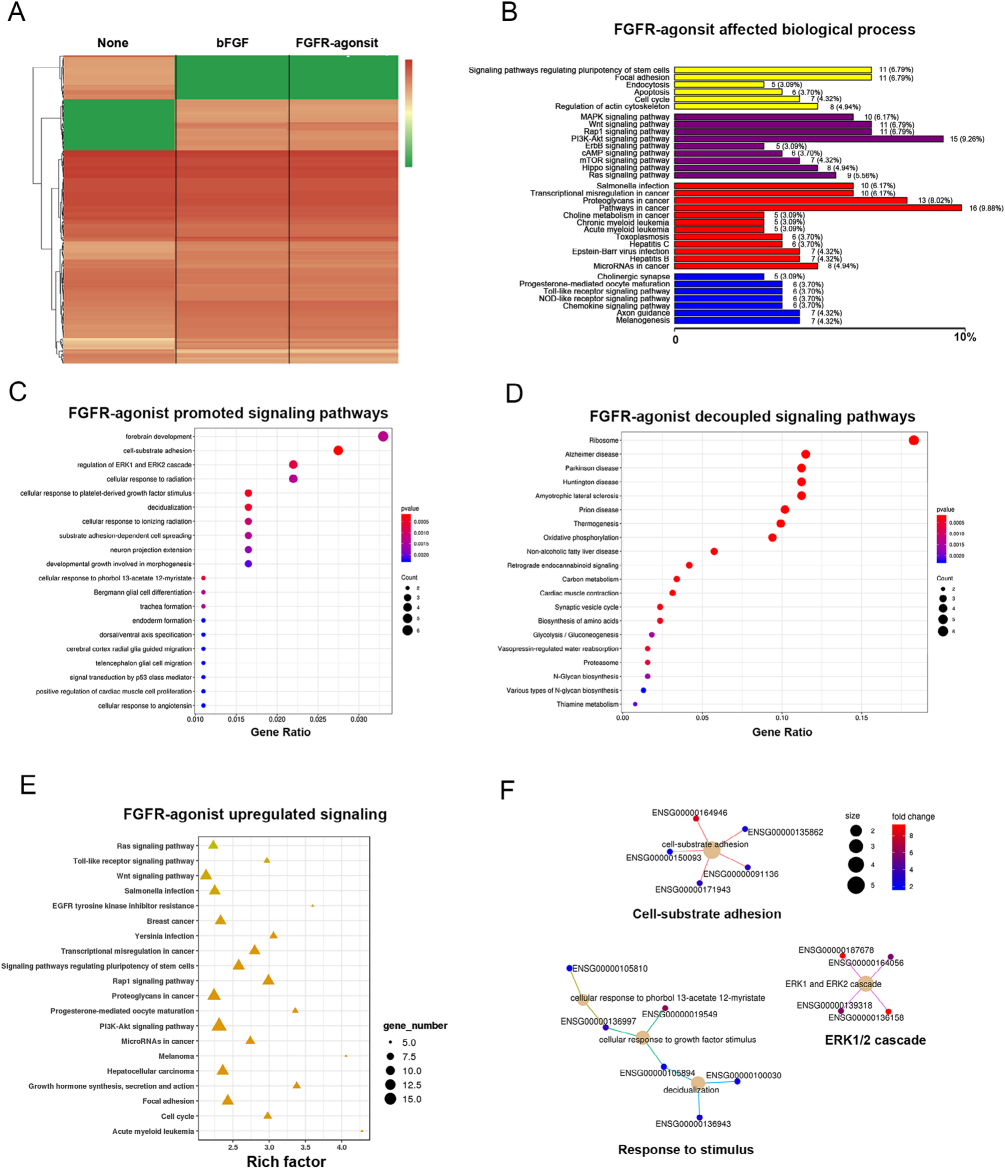
**FGFR-agonist programmed transcriptional profile of NPCs.** (**A**) Comparative gene expression heatmaps in NPCs treated with FGFR-agonist and bFGF. The heatmaps display the transcriptional profiles of NPCs exposed to FGFR-agonist or bFGF for 10 days, relative to untreated NPCs (None). Both upregulated (red) and downregulated (green) gene profiles demonstrate a high degree of similarity between the two treatment conditions. (**B**) Functional enrichment analysis of upregulated DEGs in FGFR-agonist-treated NPCs versus untreated NPCs, utilizing gene ontology (GO) terms. It displays the top 10 significantly enriched GO terms, with the corresponding number of genes in parentheses. (**C**) A KEGG pathway enrichment analysis of the upregulated DEGs in FGFR-agonist-treated NPCs compared to untreated NPCs, indicating the top 20 significantly enriched pathways, denoted by the -log10 (adjusted p-value). (**D**) A similar KEGG pathway enrichment analysis for downregulated DEGs in FGFR-agonist-treated NPCs compared to untreated NPCs shows the top 20 significantly enriched pathways. (**E**) This panel presents a pathway enrichment analysis of differentially expressed transcripts in FGFR-agonist-treated NPCs versus untreated NPCs, based on KEGG pathway classification, highlighting the top 20 significantly enriched pathways. (**F**) A KEGG pathway enrichment cnet plot for upregulated DEGs in FGFR-agonist-treated NPCs compared to untreated NPCs. The nodes symbolize KEGG pathways, and the edges signify overlapping genes between pathways. Node size corresponds to the number of genes in the pathway, while node color represents the -log10 (adjusted p-value) of enrichment

To explore the effects of the FGFR-agonist on NPCs at the transcriptomic level, we performed nanopore sequencing and conducted bioinformatic analysis on the neurospheres maintained with the FGFR-agonist and bFGF for 10 days. Total RNA was extracted from the neurospheres, and nanopore sequencing was employed to obtain long-read sequencing data capable of detecting full-length transcripts. The sequencing data were processed and analyzed to identify differentially expressed genes (DEGs) and investigate their functional significance. We first compared the transcription profiles of NPCs treated with the FGFR-agonist and bFGF, and the results showed that both upregulated and downregulated gene profiles were highly similar between the two conditions compared to the untreated NPCs (Fig. 4A). The similarity parameter of the transcription profiles between the FGFR-agonist and bFGF-treated NPCs was 0.996 (**Fig.** S13). Specifically, we identified 111 upregulated genes and 97 downregulated genes in NPCs treated with the FGFR-agonist compared to the untreated group (**Table S3**). The volcano plot of DEGs demonstrated the significant differential expression of transcripts between the FGFR-agonist-treated and the untreated groups (**Fig.** S14). Functional enrichment analysis of the upregulated DEGs revealed significant enrichment in gene ontology (GO) terms related to stem cell maintenance, including signaling pathways regulating pluripotency of stem cells, MAPK signaling pathway, Wnt signaling pathway, PI3K-Akt signaling pathway, and Hippo signaling pathway (Fig. 4B) [34]. Additionally, the upregulated DEGs showed significant enrichment in signaling pathways associated with NPC maintenance and self-renewal, such as forebrain development, regulation of ERK1 and ERK2 cascade, and cellular responses to platelet-derived growth factor stimulus (Fig. 4C). We focused on the KEGG pathway of regulating pluripotency of stem cells and found that several pivotal neural stem cell markers were upregulated in the FGFR-agonist treated group (**Fig.** S15). Notably, SOX2 and c-Myc, instrumental transcription factors in promoting self-renewal and pluripotency of embryonic or neural stem cells, were among the genes displaying upregulated expression following FGFR-agonist treatment [35, 36].

Furthermore, the KEGG pathway enrichment analysis of the downregulated genes upon FGFR-agonist treatment revealed that the top five related neuronal diseases were associated with neuronal dysfunctions, including Alzheimer’s disease, Parkinson’s disease, Huntington’s disease, and Amyotrophic lateral sclerosis. This bioinformatic data suggests the potential of the FGFR-agonist to decouple mature neuronal function (Fig. 4D). These findings highlight the potential of the FGFR-agonist in promoting and preserving the stemness of neural progenitor cells. The pathway enrichment revealed that several important signaling pathways for NPCs were upregulated, including the Ras signaling pathway, Wnt signaling pathway, PI3K-AKT pathway, and cell cycle (Fig. 4E). The KEGG pathway enrichment plot analysis of the upregulated genes identified three signaling networks related to cell-substrate adhesion, ERK1/2 cascade, and response to stimulus (Fig. 4F). These findings indicate that the FGFR-agonist can effectively mimic the function of bFGF in maintaining the stemness of NPCs. Our results suggest that the FGFR-agonist can promote stem cell maintenance, regulate cell responses to growth factor stimuli, and suppress neuronal differentiation. These findings further support the potential of the FGFR-agonist as an effective modulator of NPCs, comparable to the role of bFGF.

### FGFR-agonist maintains NPCs for long-term propagation

**Fig. 5:**
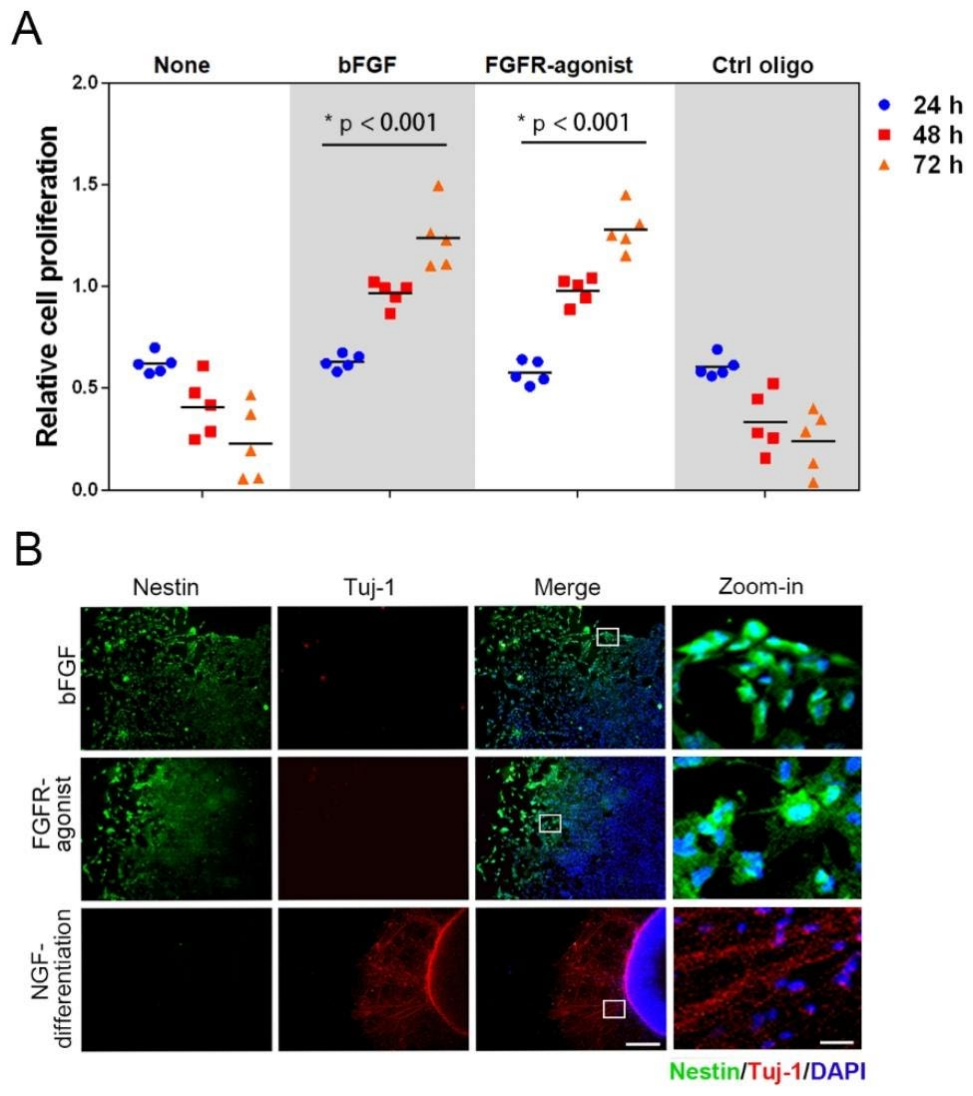
**The potency for neurogenesis of long-term propagated NPCs by FGFR-agonist.** (**A**) The influence of FGFR-agonist on proliferative capacity of the NPCs after 50 passages. NPCs maintained in FGFR-agonist were propagated for 50 passages and treated with either bFGF, FGFR-agonist or Ctrl-oligo for 72 h. The cell proliferation was assessed using the CCK8 assay. Data are depicted as mean ± SD (n = 5), with *p < 0.001 (one-way ANOVA). (**B**) Long-term propagated NPCs underwent different treatment regimens, including bFGF, FGFR-agonist, or NGF-induced differentiation, over a 10-day period. Following treatment, cells were fixed and analyzed via immunofluorescence. Nestin is represented through green fluorescence, while Tuj-1 is indicated by red fluorescence. Cell nuclei are counterstained with DAPI for contrast. Scale bar = 100 μm. Specific regions of interest (ROI), delineated by white-bordered boxes, are further magnified to provide a detailed view, accompanied by a zoom-in scale bar set at 10 μm

Next, we investigated the applicability of the FGFR-agonist for long-term maintenance of NPCs. We successfully passaged and maintained the NPCs for over 50 passages using media containing the FGFR-agonist. Throughout the passages, we did not observe any significant changes in morphology or growth characteristics of the NPCs, indicating the stability and effectiveness of the FGFR-agonist in maintaining long-term self-renewal of NPCs (Fig. 5A). The absence of both bFGF and FGFR-agonist resulted in a decrease in cell survival over time, highlighting the essential role of the FGFR-agonist in promoting cell survival and self-renewal of NPCs. Furthermore, we explored the potency of the FGFR-agonist in maintaining NPCs for neuronal differentiation. We replaced the media of the FGFR-agonist-maintained NPCs with different media containing bFGF, FGFR-agonist, or a differentiation condition. After 10 days of incubation without bFGF or FGFR-agonist, the cells exhibited elongated morphology resembling neurons and the immunofluorescence analysis revealed a significant increase in Tuj-1-positive neurons and a decrease in Nestin positive NPCs in the NGF-mediated neuronal differentiation group compared to the bFGF or FGFR-agonist treated groups (Fig. 5B). This data indicates that in the absence of FGFR-agonist, the NPCs undergo neuronal differentiation. In contrast, the FGFR-agonist treated NPCs maintained a strong staining signal for Nestin, indicating the preservation of the undifferentiated state. Together, these findings validate the functionality of the FGFR-agonist in maintaining the multipotency of NPCs for neurogenesis. Therefore, our results demonstrate that the DNA-based FGFR-agonist can serve as an effective alternative to bFGF in maintaining the stemness of NPCs while decoupling neuronal differentiation. The use of artificial agonists would provide a valuable tool for large-scale acquisition of neural cells for functional assessment, disease modeling, and potential applications in regenerative medicine.

## Discussion

The development of alternative culture systems for NPCs has been an evolving field of research, tracing back to foundational studies such as the work by Reynolds and Weiss in 1992, which first described the neurosphere culture technique [37]. Their groundbreaking study illustrated the potential of adult mammalian central nervous system cells to proliferate in vitro and differentiate into neurons and astrocytes, providing a pioneering method for maintaining neural progenitor cells in a self-renewing state. Building upon this legacy, current efforts have turned to the need for scalable and consistent NPC culture methods. One promising approach involves using a chemically defined media formulated without serum and protein growth factors. Instead, these media contain a precise combination of essential nutrients, vitamins, hormones, and signaling molecules to support NPC growth and maintenance [38, 39]. The use of the chemically defined media offers several advantages, including better control over culture conditions, reduced variability between batches, and improved reproducibility of experimental results [40]. Additionally, the elimination of serum from the culture system reduces the risk of introducing immunogenic factors, addressing concerns related to the safety and compatibility of NPCs for potential clinical applications [41]. Another strategy to overcome challenges in NPC culture is the use of small molecules and synthetic substitutes to replace protein growth factors. Small molecules can selectively activate or inhibit specific signaling pathways involved in NPC self-renewal, proliferation, and differentiation [42]. These molecules offer advantages such as increased stability, lower production costs, and improved reproducibility compared to protein growth factors [43]. For example, small molecule agonists of the Wnt signaling pathway, such as CHIR99021, have been shown to enhance NPC proliferation and maintain their undifferentiated state [44]. Similarly, small molecule inhibitors of the transforming growth factor-beta (TGF-β) signaling pathway, like SB431542, can promote NPC differentiation into specific neural cell lineages [45, 46]. In addition to small molecules, synthetic substitutes for protein growth factors, such as DNA-based aptamers, have emerged as promising alternatives. Aptamers are short single-stranded DNA molecules that can be engineered to specifically bind and activate cell surface receptors, mimicking the effects of protein ligands [47]. These aptamers offer advantages such as high specificity, stability, and ease of synthesis [48]. By designing aptamers that target key receptors involved in NPC self-renewal and differentiation, researchers can develop innovative strategies to modulate NPC behavior and culture them more efficiently.

In this study, we explored the potential of a DNA-based modulator as an alternative to protein growth factors for the maintenance and expansion of human-derived NPCs. Our focus was on the FGF/FGFR signaling pathway, which plays a crucial role in NPC self-renewal and differentiation [29]. By designing a DNA-based agonist specific to FGFR1, we aimed to activate downstream signaling and promote NPC stemness while overcoming the limitations associated with bFGF [49]. To evaluate the effectiveness of our DNA-based modulator, we conducted a comprehensive analysis of agonist-mediated FGFR signaling and transcriptome changes in NPCs. This analysis allowed us to assess the impact of the DNA-based modulator on long-term NPC self-renewal in culture. The successful implementation of our DNA-based modulator holds immense potential for disease modeling, drug screening, and regenerative medicine applications. The potential of DNA aptamer-derived constructs as synthetic substitutes for bFGF in the culture of HESC and human induced pluripotent stem cells (iPSCs) has been previously explored. Notably, the Sando group made a significant contribution by engineering a 76-mer single-stranded DNA aptamer, capable of supporting the self-renewal and pluripotency of iPSCs and also provided a comprehensive protocol for feeder-free hiPSC maintenance using a DNA aptamer-based mimic of bFGF [30, 50]. Building upon this pioneering work, our current study further expands by applying the aptamer-based strategy to NPCs and demonstrate the potential of DNA-based FGFR-agonist to endow the NPCs with long-term pluripotency over 50 passages, offering a consistent and scalable method for culturing NPCs. We found that our DNA-based FGFR-agonist effectively activates downstream signaling pathways and maintains the self-renewal and pluripotency of NPCs, performing as well as or even better than bFGF in these regards. This brings into focus the DNA-based FGFR-agonist as not just an alternative, but potentially a superior substitute to bFGF for maintaining and culturing NPCs. Given its greater stability, scalability, and economic advantages, our DNA-based agonist holds immense potential for revolutionizing the field of NPC culture.

In light of our promising findings, it is worth mentioning that our study is not without limitations that warrant further investigation. One such limitation is the absence of karyotype testing to assess the genomic stability of NPCs maintained under long-term culture with our DNA-based FGFR-agonist. The significance of genomic stability cannot be understated, especially when contemplating the therapeutic applications or in-depth biological studies of these cells. We recognize the importance of such tests in assessing the genomic stability of cells maintained under long-term culture and consider it a pivotal area for future research. Moreover, our study has not employed control groups using FGFR antagonists or specific inhibitors. Including such controls could shed light on the specificity and mechanism of action of our DNA-based agonist and could serve to more robustly validate its function. We acknowledge this as an important avenue for future investigations to offer a more nuanced understanding of the interactions between our agonist and FGFR signaling pathways.

Our work might present new opportunities for studying the pathology of neurological disorders, identifying potential therapeutic targets, and screening drug candidates, thereby broadening the scope of applications in neuroscience and regenerative medicine. [51]. The ability to generate large quantities of high-quality NPCs might facilitate the development of cell-based therapies to replace or repair damaged neural tissue [52]. One of the most compelling arguments for the use of our DNA-based agonist is its economic advantage. bFGF, a protein-based growth factor, has relatively high production and storage costs. In contrast, DNA-based therapeutics offer advantages in production cost, accessibility, and scalability. DNA-based modulators can be synthesized, modified, and optimized for specific receptor interactions, providing flexibility and customization [30]. They also offer greater stability and reproducibility, reducing batch-to-batch variability and experimental inconsistencies [53]. Our preliminary work sheds light on the advantages and feasibility of using nucleic acid agonists as an alternative to bFGF, providing valuable insights for the development of future culture systems and their practical applications in neuroscience and regenerative medicine [54]. In summary, our findings offer a more consistent, scalable, and cost-effective method for the large-scale production of NPCs by addressing the limitations of current culture systems. The successful implementation of our DNA-based modulator holds immense potential for disease modeling, drug screening, and regenerative medicine applications, paving the way for future advancements in neuroscience research and therapeutic development.

## Materials and methods

### Materials

RPMI1640 and DMEM culture media were procured from BasalMedia Biotechnology Co., Ltd (Shanghai, China). Fetal bovine serum (FBS) was sourced from Biological Industries (USA). Serum-free DMEM/F12, KnockOut Serum Replacement (KOSR), nonessential amino acids, 110 µM 2-Mercaptoethanol, N2, B27, Dorsomorphin, SB431542, the recombinant bFGF protein and Nerve Growth Factor (NGF) were purchased from Thermo Fisher Scientific. mTeSR1 medium was obtained from Stem Cell Technologies. The Matrigel-coated plates (adhesive) were from Corning The ECL substrate solution, cell freezing solution, Penicillin-Streptomycin (100X), 0.25% Trypsin-EDTA (1X) and 3-(4, 5-dimethyl-2-thiazolyl)-2,5-diphenyl-2-H-tetrazolium bromide (CCK-8) were purchased from New Cell & Molecular Biotech Co., Ltd. The extracellular domain of human FGFR1 protein was obtained from Sino Biological. The primary antibodies for phospho-ERK1/2 (T202/Y204) and p-FGFR1(Tyr653/654) were obtained from Cell Signaling Technology (Massachusetts, USA). The antibodies for Nestin, Tuj-1, Pax6 and MAP2 were obtained from Abcam (Massachusetts, USA). Phosphorylated FGFR1 (Tyr653/654) ELISA kit was from R&D Systems. The primary antibodies of α-tubulin and GAPDH were purchased from Cellway Biotechnology Co., Ltd (Changsha, China). The secondary antibodies, including goat anti-rabbit IgG (H&L)-HRP and goat anti-mouse IgG (H&L)-HRP, were obtained from Invitrogen. The phosphatase and protease inhibitors were purchased from Topscience Co., Ltd (Shanghai, China). The nitrocellulose membrane was obtained from Merck Millipore (Darmstadt, Germany). All oligonucleotide sequences including the FAM fluorophore-conjugated FGFR-binder or FGFR-agonist were synthesized by Sangon Biological Engineering Technology & Co., Ltd (Shanghai, P. R. China) and purified using high-performance liquid chromatography. The oligonucleotides were dissolved in phosphate-buffered saline (PBS) at a pH of 7.4 and a concentration of 10 µM, then stored at -20 °C for further use.

### Native polyacrylamide gel electrophoresis (PAGE)

An 8% native polyacrylamide gel was prepared and pre-run for 30 min at 100 V. FGFR-agonist, FGFR-binder, and a control oligonucleotide (Ctrl oligo) were diluted to 500 nM, heated to 95 °C for 3 min, and then gradually cooled to room temperature. 20 µL of each sample was loaded onto the gel. Electrophoresis was performed at 100 V for 1.5-2 h at 4 °C. After electrophoresis, the gel was stained with SYBR Gold Nucleic Acid Gel Stain (Invitrogen) for 30 min and the stained gel was visualized using a UV transilluminator, and images were captured for analysis.

### Surface Plasmon Resonance (SPR) analysis

The binding kinetics of the DNA aptamer to the extracellular domain of FGFR-1 were assessed using an SPR instrument (Biacore T200). The extracellular domain of human FGFR1 protein was immobilized on a CM5 sensor chip via standard amine coupling. The DNA aptamer, diluted in running buffer (HBS), was flowed over the chip at concentrations ranging from 0.1 to 10 nM. Real-time binding curves were analyzed to determine the association (k_a_) and dissociation (k_d_) rates, from which the equilibrium dissociation constant (KD) was calculated.

### Cell binding affinity analysis

NIH3T3 cells were cultured in DMEM supplemented with 10% fetal bovine serum (FBS), 1% penicillin/streptomycin, and maintained at 37 °C in a humidified atmosphere with 5% CO2. For serum starvation, cells were washed twice with phosphate-buffered saline (PBS) and incubated in serum-free DMEM for 24 h. For flow cytometry, NIH3T3 cells (1 × 10^5^) were prepared as cell suspensions in cell dissociation buffer consisting of Hank’s Balanced Salt Solution (HBSS) supplemented with 2 mM EDTA and 0.25% trypsin. Cells were incubated with the FAM fluorophore-conjugated FGFR-binder or FGFR-agonist with varying concentrations (0, 10, 50, 100, 200, 400, 600, 800 nM) in 100 µL of flow cytometry buffer consisted of PBS containing 1% bovine serum albumin (BSA) and 0.1% sodium azide at room temperature. Subsequently, the cells were washed twice with 200 µL of PBS at room temperature. The cells were suspended in 200 µL of flow cytometry buffer and subjected to flow cytometry analysis using a flow cytometer (BD Accuri TM C6 Plus, USA). The fluorescence intensity of the cell population was then quantified using a flow cytometer, specifically evaluating the mean fluorescence intensity (MFI) as an indirect measure of ligand-receptor binding affinity. MFI values were plotted against varying concentrations of the ligand to generate a binding curve. The dissociation constant (KD) was calculated from the curve using nonlinear regression analysis, with lower KD values indicating higher affinity.

### Human embryonic stem cell culture

The human embryonic stem cell line H9 (HESCs) obtained from Cellway Bio was maintained in a standard hESC/hESCs medium. The medium consisted of DMEM: F12 supplemented with 20% KnockOut Serum Replacement (KOSR) and 20 ng/ml basic fibroblast growth factor (bFGF) from Invitrogen. Additional components included 1X nonessential amino acids and 110 µM 2-Mercaptoethanol. The HESCs (1 × 10^4^) were cultured on Matrigel-coated plates using mTeSR1 medium at 37℃. The culture plates were passaged every 6–8 days, and fresh mTeSR medium was replaced daily to maintain the cells. On the 5 days after passaging the HESCs, the cells were rinsed with sterile PBS, and a small amount of embryoid body differentiation medium was added. The HESCs colonies were gently scraped using a sterile cell scraper to detach them from the culture plate and suspend them in the medium. The suspended HESC colonies were then transferred to cell culture dishes and cultured in the differentiation medium at 37℃ for at least 5 days. Fresh medium was replaced every 2 days during this period to support the differentiation process. To preserve the HESCs, they were stored in a cell freezing medium, ensuring their long-term viability and use in future experiments.

### Preparation of neural progenitor cells (NPCs)

After 10 days of suspension culture, the hESCs formed uniform embryoid bodies with similar structures, that are crucial for the formation of neurospheres. We applied a technique pioneered by Reynolds and Weiss in 1992 for the propagation of neural stem and progenitor cells [54]. The differentiation medium was then replaced with a mixture of N2 (1X), B27(2%), and serum-free DMEM/F12 supplemented with Dorsomorphin (2 µM) and SB431542(10 µM). Subsequently, the embryoid bodies were transferred to Matrigel-coated dishes and cultured at 37 °C for 10 days. Fresh N2/B27 medium supplemented with 10 ng/mL bFGF replacement was provided every 2 days. After 6 to 10 days, the cells located in the center of the embryoid bodies underwent differentiation, acquiring rosette-like structures characteristic of NPCs. To collect the NPCs, a sterile needle was carefully used to aspirate the cells, which were then transferred to a sterile centrifuge tube. To facilitate cell pelleting, DMEM/F12 serum-free medium equivalent to 2/3 of the tube volume was added, and the cells were centrifuged at 1000 rpm for 5 min. The resulting cell pellet was resuspended in a mixture of N2 (1X), B27 (2%) and serum-free DMEM/F12 supplemented with 10 ng/mL bFGF. The NPCs (1 × 10^5^) were harvested and maintained in the NPC culture medium, which consisted of either protein-based bFGF or DNA-based FGFR-agonists. In the following study, unless otherwise specified for experiments conducted beyond 50 passages, NPCs were used at a passage number less than 10 at the initiation of the experiments. For the subsequent stage of mature neuronal differentiation, N2 (1X) and B27 (2%) medium containing NGF (50 ng/mL) was utilized, and the NPCs were cultured for an additional 10 to 14 days. This culture condition supported the NPCs in differentiating into mature neurons.

### Immunofluorescence staining

To assess stemness and neuronal differentiation, we conducted immunofluorescence staining experiments. Cells were fixed with 4% paraformaldehyde and permeabilized with 0.1% Triton X-100. After blocking in 5% bovine serum albumin (BSA), cells were incubated with primary antibodies against MAP2 (200 dilution) or Tuj-1 (500 dilution) to identify neurons, as well as Nestin (500 dilution) and Pax 6 (300 dilution) to identify neural progenitor cells. Following primary antibody incubation, cells were washed and then incubated with secondary antibodies: Alexa Fluor 488-conjugated rabbit secondary antibody (500 dilution), and Alexa Fluor 594-conjugated mouse secondary antibody (500 dilution). Confocal laser scanning microscopy (CLSM) (Nikon, Eclipse TE2000-E, Japan) was used to capture images of the stained cells from 5 random fields per sample, and each experiment was repeated three times. Image analysis and quantification were performed using ImageJ software.

### ELISA method to study FGFR phosphorylation

To evaluate the activation of FGFR1, serum-starved cells (1 × 10^6^) were stimulated with FGFR-agonist at a concentration of 40 nM or with bFGF at a concentration of 20 ng/mL for a period of 10 min at 37 °C. Phosphorylation of FGFR1 at Tyr653/654 was quantified using an ELISA kit (R&D Systems) according to the manufacturer’s instructions. Briefly, 100 µl of each cell lysate was added to wells of an ELISA plate pre-coated with anti-p-FGFR1 (Tyr653/654) antibodies. Following incubation and washing steps, a secondary antibody conjugated with horseradish peroxidase (HRP) was added. The reaction was developed using a chromogenic substrate and stopped with the addition of a stop solution. The absorbance was measured at 450 nm using a microplate reader, and the phosphorylation levels were calculated based on a standard curve and normalized to the untreated group.

### Western blot analysis

To investigate the activation of the ERK signaling pathway in response to FGFR-agonist and bFGF treatment, NIH3T3 cells or NPCs (1 × 10^5^) were subjected to serum starvation. Following starvation, cells were stimulated with either 40 nM FGFR-agonist or 20 ng/mL bFGF for a period of 10 min at 37 °C. Cells were then lysed in RIPA buffer containing protease and phosphatase inhibitors to extract proteins. Equal amounts of protein samples were loaded into SDS-PAGE gels for electrophoresis. After separation, proteins were transferred onto a polyvinylidene fluoride (PVDF) membrane. The membrane was blocked with 5% non-fat milk or BSA in TBS-T (Tris-buffered saline with Tween 20) for 1 h at room temperature. Primary antibody incubation was performed overnight at 4 °C using antibodies specific to phospho-ERK1/2 (Thr202/Tyr204), total ERK1/2, and GAPDH as a loading control. Following extensive washing with TBS-T, the membrane was incubated with appropriate HRP-conjugated secondary antibodies for 1–2 h at room temperature. The membrane was washed again before the chemiluminescent substrate was applied. Signal detection and capture were performed using a multifunctional molecular imaging system (Azure Biosystems 600, USA). The band intensities were quantified using ImageJ software to analyze the phosphorylation levels of ERK1/2.

### Neurosphere formation and assessment

NPCs (1 × 10^5^) were cultured in a suspension system to form neurospheres. These NPCs were at a passage number less than 10 during the initiation of the experiment. The culture medium contained either a standard concentration of bFGF (20 ng/mL) or one of two concentrations of FGFR-agonist (40 nM or 200 nM). Cells were incubated at 37 °C in a 5% CO2 atmosphere for a duration of 10 days. To evaluate the efficiency and quality of neurosphere formation, multiple metrics were assessed, including the number, size, and morphology of neurospheres. Phase-contrast images were captured every day for 10 days using an inverted microscope. ImageJ software was utilized for the analysis of neurosphere characteristics. To estimate the volume of the neurospheres, a standard 100 μm scale bar was used for calibration. The perimeter and area of each neurosphere were measured using the ImageJ. The volume V of the neurospheres was estimated using the formula: $V=\frac{4}{3}\times \pi \times {\left(\frac{d}{2}\right)}^{3}$, while *d* is the diameter.

### Proliferation assay

NPCs proliferation in the presence of FGFR-agonist or bFGF was determined using a Cell Counting Kit-8 (CCK-8) assay. NPCs were seeded in a 96-well plate at a density of 1 × 10^5^ NPCs per well and incubated for 24 and 72 h. Following the respective incubation periods, the CCK-8 reagent was added to each well, and the absorbance at 450 nm was measured using a microplate reader. The CCK-8 assay provides a quantitative assessment of cell viability and proliferation by measuring the metabolic activity of the cells, with higher absorbance values indicating increased cell proliferation.

### Transwell assay

To evaluate the migratory behavior of NPCs, a Transwell migration assay was performed using 24-well Transwell inserts with 8.0 μm pore polycarbonate membranes. NPCs (1 × 10^6^) were harvested and resuspended in a serum-free culture medium before being placed in the upper chamber. The lower chamber was filled with 600 µL of culture medium containing either bFGF at a concentration of 20 ng/mL or FGFR-agonist at 40 nM to serve as chemoattractants. After 24 h incubation, non-migrated cells were removed from the upper membrane surface, while the migrated cells on the lower surface were fixed and stained with 0.1% w/v crystal violet. Phase-contrast images were captured using an optical microscope. For quantitative analysis, the crystal violet staining was solubilized, and the optical density of each well was measured. The stained area was then normalized to the bottom surface area using ImageJ software. The assay was performed in six biological replicates for robust quantitative analysis, and the data were subsequently used for statistical analysis.

### Transcriptomic analysis

Total RNA was isolated from NPC monolayers (1 × 10^7^) that had been treated with either bFGF at a concentration of 20 ng/mL or an FGFR-agonist at 40 nM for 10 days. RNA extraction was performed using the TRIzol reagent (Invitrogen), strictly following to the manufacturer’s guidelines. Subsequent to RNA extraction, the samples underwent RNA sequencing analysis on an Oxford Nanopore Technologies platform. The generated sequencing data were processed and analyzed using a customized bioinformatics pipeline. To gain insights into the functional significance of the differentially expressed genes (DEGs), gene ontology (GO) analysis and Kyoto Encyclopedia of Genes and Genomes (KEGG) pathway analysis were performed. GO analysis provided information on the biological processes, cellular components, and molecular functions associated with the DEGs. KEGG pathway analysis identified the signaling pathways and networks in which the DEGs were significantly enriched. These analyses aimed to identify genes and pathways involved in stem cell maintenance and neural differentiation. The results of the bioinformatics analysis provided valuable insights into the molecular mechanisms underlying the effects of artificial FGFR-agonist and bFGF on stem cell maintenance and neural differentiation.

### Long-term NPCs maintenance and neuronal differentiation

To assess the differentiation potential of NPCs cultured in the presence of the FGFR-agonist or bFGF, the cells were passaged and maintained for over 50 passages. Subsequently, the cells were subjected to a differentiation assay by withdrawing bFGF or the FGFR-agonist and replacing it with a neuronal differentiation medium. After 10 days of differentiation, the cells were fixed and immunostained with specific antibodies against neuronal marker Tuj-1 (Abcam) and NPC marker Nestin (Abcam). Fluorescence microscopy analysis was performed to visualize and evaluate the presence of differentiated neurons (Tuj-1-positive cells) and the maintenance of neural progenitor cell characteristics (Nestin-positive cells) in the cultures. Confocal laser scanning microscopy (CLSM) (Nikon, Eclipse TE2000-E, Japan) was used to acquire images of the stained cells. The immunostaining results provided insights into the effect of the FGFR-agonist compared to bFGF in promoting neuronal differentiation and maintaining the NPC phenotype.

### Statistical analysis

All experiments were conducted with triplicate samples, and the data are presented as the mean ± standard deviation (SD). Statistical analyses were performed using unpaired two-tailed Student’s t-test or one-way analysis of variance (ANOVA), followed by Tukey’s post hoc test, as appropriate. A p-value of less than 0.001 was considered statistically significant. Statistical significance is indicated as follows: *p < 0.01, **p < 0.001. If the p-value exceeds 0.05, it is indicated as “n.s.“ (no significance).

### Electronic supplementary material

Below is the link to the electronic supplementary material.

Supplementary Material 1

## Data Availability

All data supporting the findings of this study are available within the article and its supplementary information files. Source data are provided in this paper. Any other relevant data or reagents are available from the corresponding authors upon reasonable request.

## Declarations

### Authors’ information

Dr. Hong-Hui Wang.

Position: Associate Professor.

Affiliation: College of Biology, Hunan University, Changsha, China.

Research Interests: Cell signaling, DNA nanotechnology, and pharmacology.

Publications: Over 40 articles in peer-reviewed journals.
