# Supplementary material for: Development of synthetic modulator enabling long-term propagation and neurogenesis of human embryonic stem cell-derived neural progenitor cells
Source: Biol Res. 2023 Nov 11;56:59. doi: 10.1186/s40659-023-00471-0 (PMC10638775; doi:10.1186/s40659-023-00471-0)
Supplement: Supplementary file 1 — Supplementary Material 1 [file 40659_2023_471_MOESM1_ESM.docx]

**Supplemental Information**

**Development of Synthetic Modulator Enabling Long-Term Propagation and Neurogenesis of Human Embryonic Stem Cell-Derived Neural Progenitor Cells**

Ceheng Liao^1^#, Ying Guan^2^#, Jihui Zheng^1^, Xue Wang^1^, Meixia Wang^1^, Zhouhai Zhu^2^, Qiyuan Peng^2^, Hong-Hui Wang^1^*, and Meng Li^2^*

^1^College of Biology, Hunan University, 27 Tianma Road, Yuelu District, Changsha, Hunan 410082, China

^2^Joint Institute of Tobacco and Health, 367 Hongjin Road, Wuhua District, Kunming, Yunnan 650202, China

#

Corresponding author

*Hong-Hui Wang, wanghonghui@hnu.edu.cn

*Meng Li, mengli.kib@gmail.com

**Supplemental Methods**

**Wound scratch assay**

Cells (1x105) were seeded in a 12-well plate at a density that allowed them to reach approximately 70-80% confluence as a monolayer after 24 hours of incubation. Subsequently, wounds were created in each cell monolayer by carefully scratching them using a sterile 200 μL pipette tip. After scratching, the wells were gently washed twice with the culture medium to remove detached cells. The cells were then incubated with either bFGF or FGFR-agonist. The rate of wound healing was monitored and imaged at 24 and 48 hours using a Cytation 5 microplate reader equipped with a positioning system. The images captured at different time points were analyzed to measure the extent of wound closure, providing quantitative data on the rate of cell migration and wound healing.

**Measurement of circularity of neurosphere**

NPCs were cultured in a suspension system to form neurospheres as described in the manuscript. Phase-contrast images were captured at day 7 using an inverted microscope. ImageJ software was utilized for the analysis of neurosphere characteristics. The circularity of neurosphere was measured using ImageJ software, according to a step-by-step guide for measuring the circularity of neurosphere images. The formula used for calculating circularity is: $Circularity=4\times\pi\times\frac{Area}{{Perimeter}^{2}}$. The perimeter and area of each neurosphere were measured using the ImageJ. A circularity value close to 1 indicates a more circular shape, while values deviating from 1 suggest irregular shapes.

**Table S1. The sequences of oligonucleotides**

| Name | Sequence (5’---3’) |
| --- | --- |
| FGFR-binder | GCC GCG TCT TTA TGG CTG GGG ATG GTG TGG GTT GCG GC |
| FGFR-agonist | GCC GCG TCT TTA TGG CTG GGG ATG GTG TGG GTT GCG GCG CCG CGT CTT TAT GGC TGG GGA TGG TGT GGG TTG CGG C |
| Ctrl oligo | CGG CGT TGG GTG TGG TAG GGG TCG GTA TTT CTG CGC CGC GGC GTT GGG TGT GGT AGG GGT CGG TAT TTC TGC GCC G |

**Table S2. The kinetic parameters of FGFR1 binding performance of FGFR-agonist in SPR assay (fitting the data to the1:1 binding model)**

| Name | K_a_ (1/Ms) | K_d_ (1/s) | KD (M) |
| --- | --- | --- | --- |
| FGFR-binder | 2.20E+10 | 5.49E+01 | 2.50E-09 |
| FGFR-agonist | 4.51E+06 | 2.08E-04 | 4.61E-11 |

**Table S3. Summary of the number of differentially expressed genes (DEGs)**

| **Samples** | **DEGs** | **Upregulated** | **Downregulated** |
| --- | --- | --- | --- |
| None vs bFGF | 189 | 75 | 114 |
| None vs FGFR-agonist | 208 | 111 | 97 |


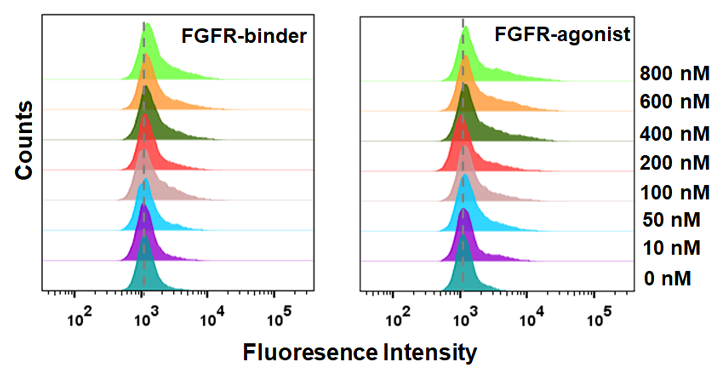


**Fig. S1. Examination of FGFR-binder and FGFR-agonist binding affinity with NIH3T3 cells via flow cytometry.**


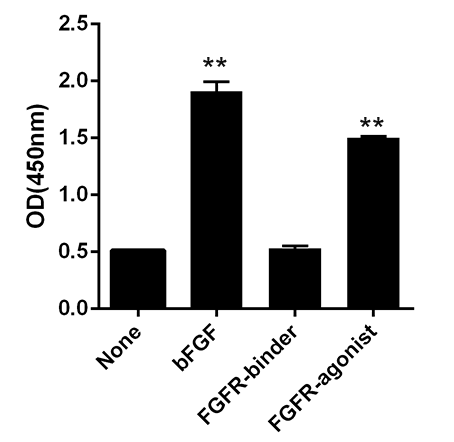


**Fig. S2. FGFR-agonist promotes FGFR phosphorylation in NIH3T3 cells.** The FGFR phosphorylation (Tyr653/654) in NIH3T3 cells in response to different treatments (untreated, bFGF, FGFR-binder and FGFR-agonist was assessed using an ELISA kit. All data are presented as means ± SD from three independent experiments. **p < 0.001 (unpaired two-tailed Student’s t-test).


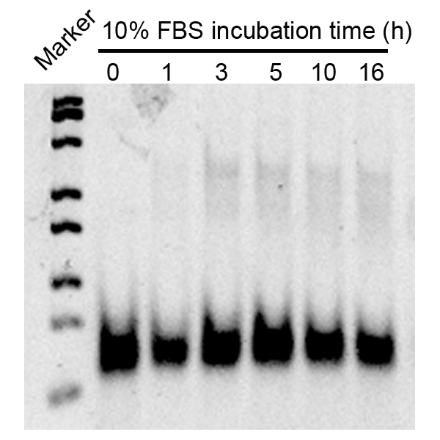


**Fig. S3. Serum stability assessment of FGFR-agonist.** The DNA oligonucleotide of FGFR-agonist were incubated with 10% fetal bovine serum (FBS) for varying durations, followed by PAGE gel electrophoresis to evaluate stability.

**
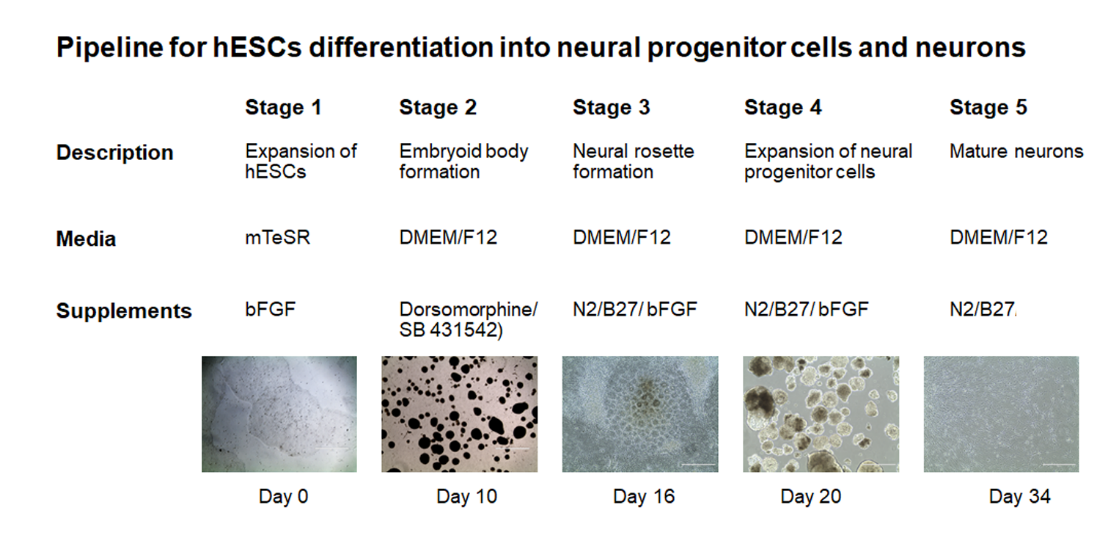
**

**Fig. S4. Schematic diagram of the experimental procedure for neuronal differentiation from hESCs.** The necessary supplements and media for each stage are listed. Representative images of hESCs (Stage 1), Embryoid Body (Stage 2), Neural Rosette (Stage 3), Neural Progenitor Cells (Stage 4), and Mature Neurons (Stage 5) were captured under light microscopy at indicated time points. Scale bar: 500 μm.

**
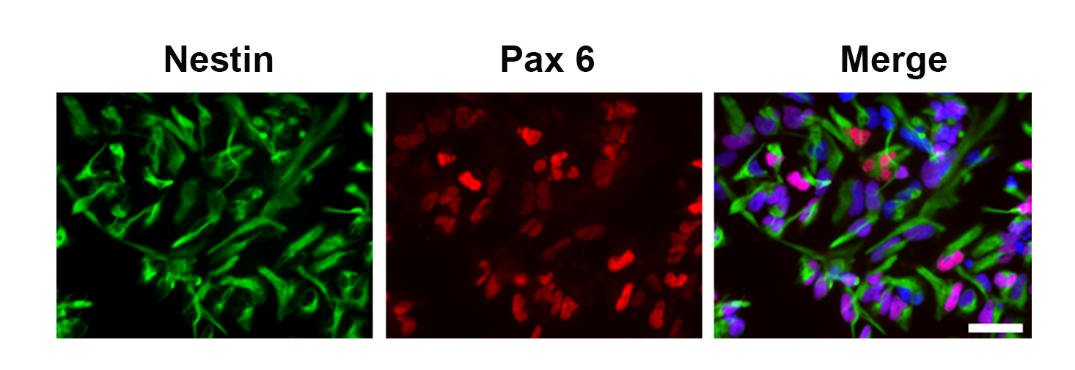
**

**Fig. S5. Immunofluorescence staining of hESC-Derived NPCs.** Cells were fixed and stained with antibodies against Nestin (Green) and Pax 6 (Red). Nuclei were counterstained with DAPI (Blue). Scale bar: 50 μm.

**
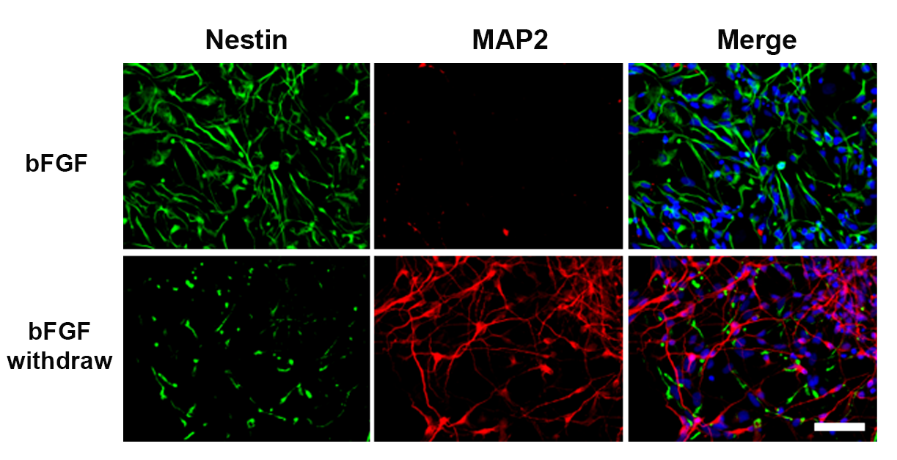
**

**Fig. S6**. **Immunofluorescence staining of NPCs following 10-day treatment with or without bFGF.** NPCs were fixed and stained for Nestin (Green) and MAP2 (Red). Nuclei were counterstained with DAPI (Blue). Scale bar: 50 μm.


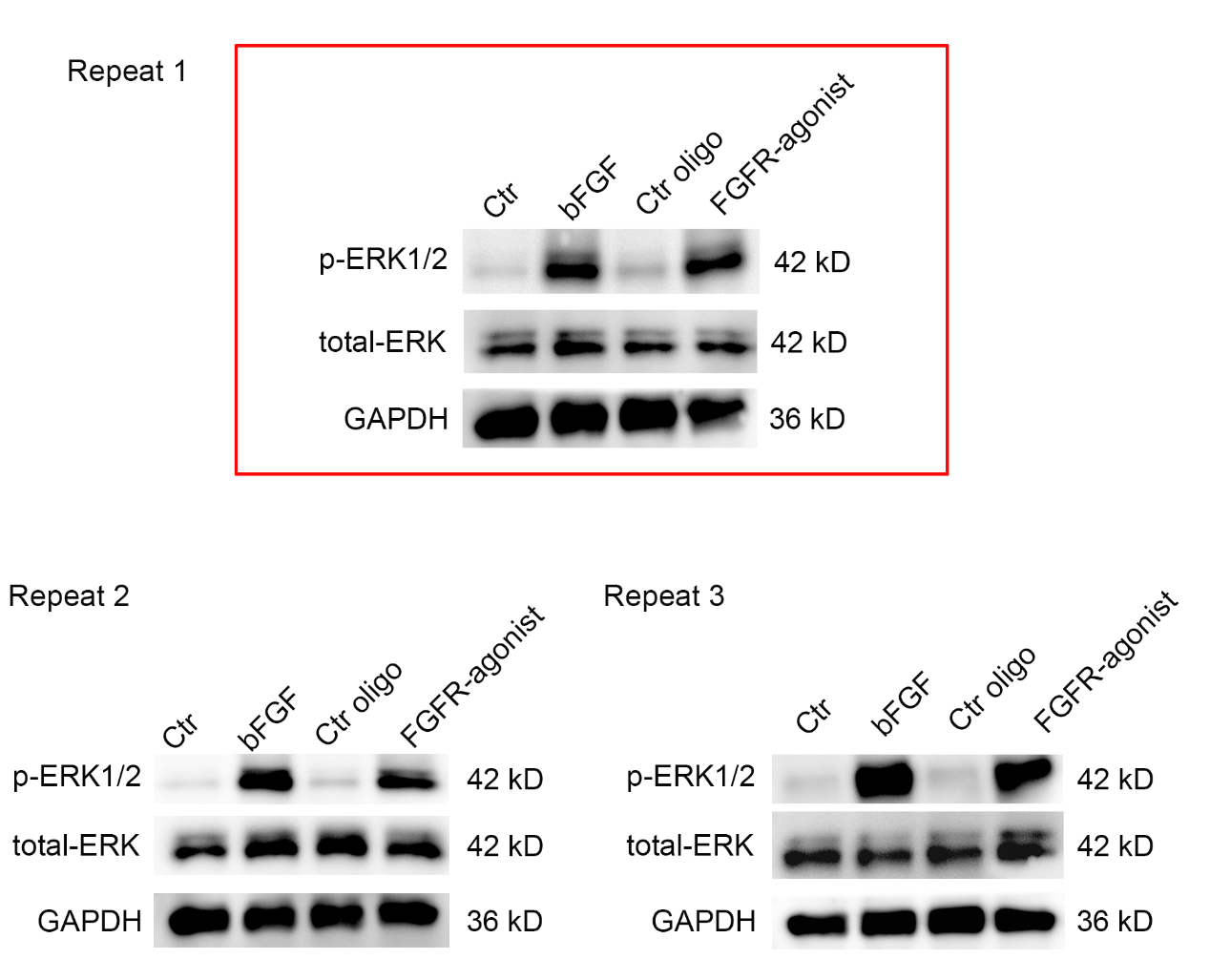


**Fig. S7. FGFR-agonist Enhances ERK1/2 Phosphorylation in NPCs.** Western blot analyses were carried out to assess the phosphorylation levels of ERK1/2 at residues Thr202/Tyr204. Serum-starved NPCs were exposed to either bFGF (20 ng/mL) or FGFR-agonist (40 nM) for 10 minutes. The phosphorylation status of ERK1/2 and the levels of total ERK were then probed via Western blot, using GAPDH as an internal loading control. To ensure the reliability of the quantitative measurements, the experiment was performed in triplicate. The data thus obtained were subsequently subjected to statistical analyses, which are depicted in **Fig. 2C**. Areas highlighted by red boxes indicate the selected data used to generate the representative blot shown in **Fig. 2B**.


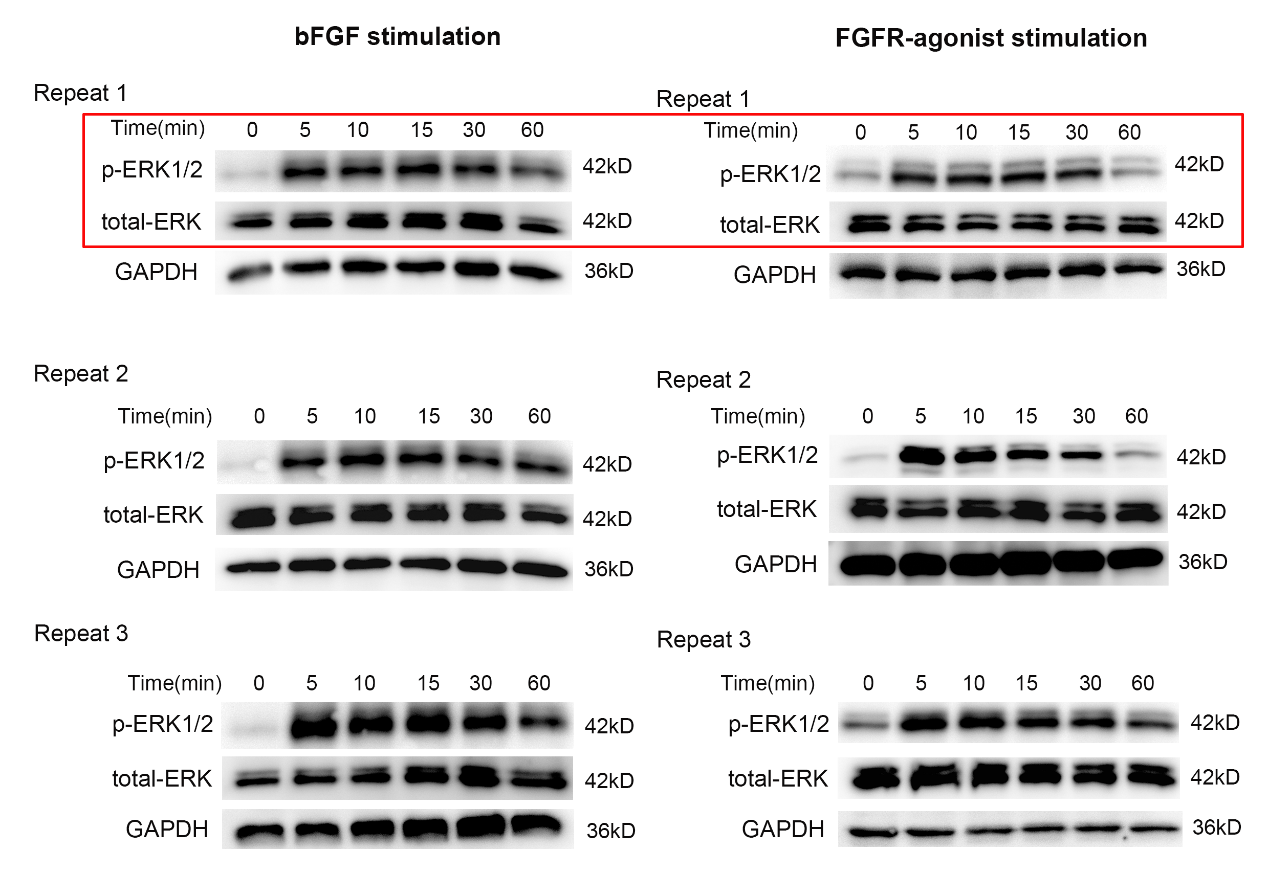


**Fig. S8**. **The kinetics of ERK1/2 phosphorylation of NPCs after FGFR-agonist stimulation**. Serum-starved NPCs were exposed to either bFGF (20 ng/mL) or FGFR-agonist (40 nM) over varying time intervals (5, 10, 15, 30, 60 minutes). Phosphorylation of ERK1/2 (Thr202/Tyr204) and the levels of total ERK was examined by Western blot, with GAPDH serving as an internal control. The experiment was performed in triplicate to ensure quantitative robustness, and the data were subsequently used for the statistical analysis presented in Fig. **2E**. Areas highlighted by red boxes indicate the selected data used to generate the representative blot shown in **Fig. 2D**.

**
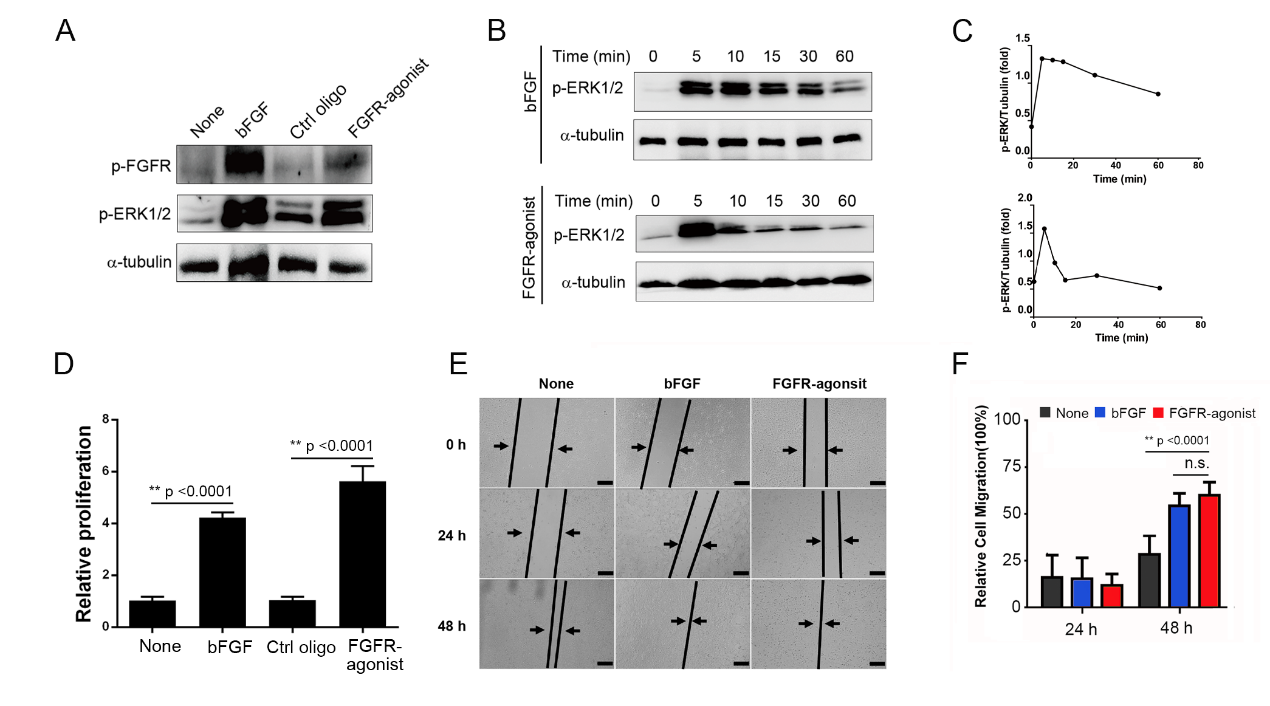
**

**Fig. S9. FGFR-agonist promotes FGFR signaling and cellular behaviors of ATDC cells. (**A) Assessment of p-FGFR1(Tyr653/654) and p-ERK1/2 (Thr202/Tyr204) levels in ATDC5 cells through Western blotting, following treatment with either bFGF or FGFR-agonist. (B) Analysis of the time-dependent activation of p-ERK1/2 in ATDC5 cells after exposure to bFGF (20 ng/mL) or FGFR-agonist (40 nM) over different time intervals (5, 10, 15, 30, 60 min). (C) Quantitative analysis was performed using Image J software to represent the kinetics of ERK1/2 phosphorylation in response to different stimulations. (D) ATDC5 cell proliferation under various conditions (None, bFGF, Ctrl oligo, FGFR-agonist), with continuous culture for 48 h. Cell proliferation was measured using the Cell Counting Kit-8 (CCK8). Data are presented as mean ± SD (n = 5), with **p < 0.001 (unpaired two-tailed Student’s t-test). (E) Examination of the impact of FGFR-agonist on ATDC5 cell migration. Cells were treated with bFGF, FGFR-agonist, or FGFR-binder for two days. Wound-closure events were tracked using light microscopy, with images captured at 0, 24, and 48 h. Scale bar = 100 μm. (F) The relative wound closure rates under various experimental conditions were quantified and normalized to establish relative cell migration compared to the areas at the start points. Data are presented as means ± SD (n = 3), with **p < 0.001 and n.s. indicating no significance (unpaired two-tailed Student’s t-test).


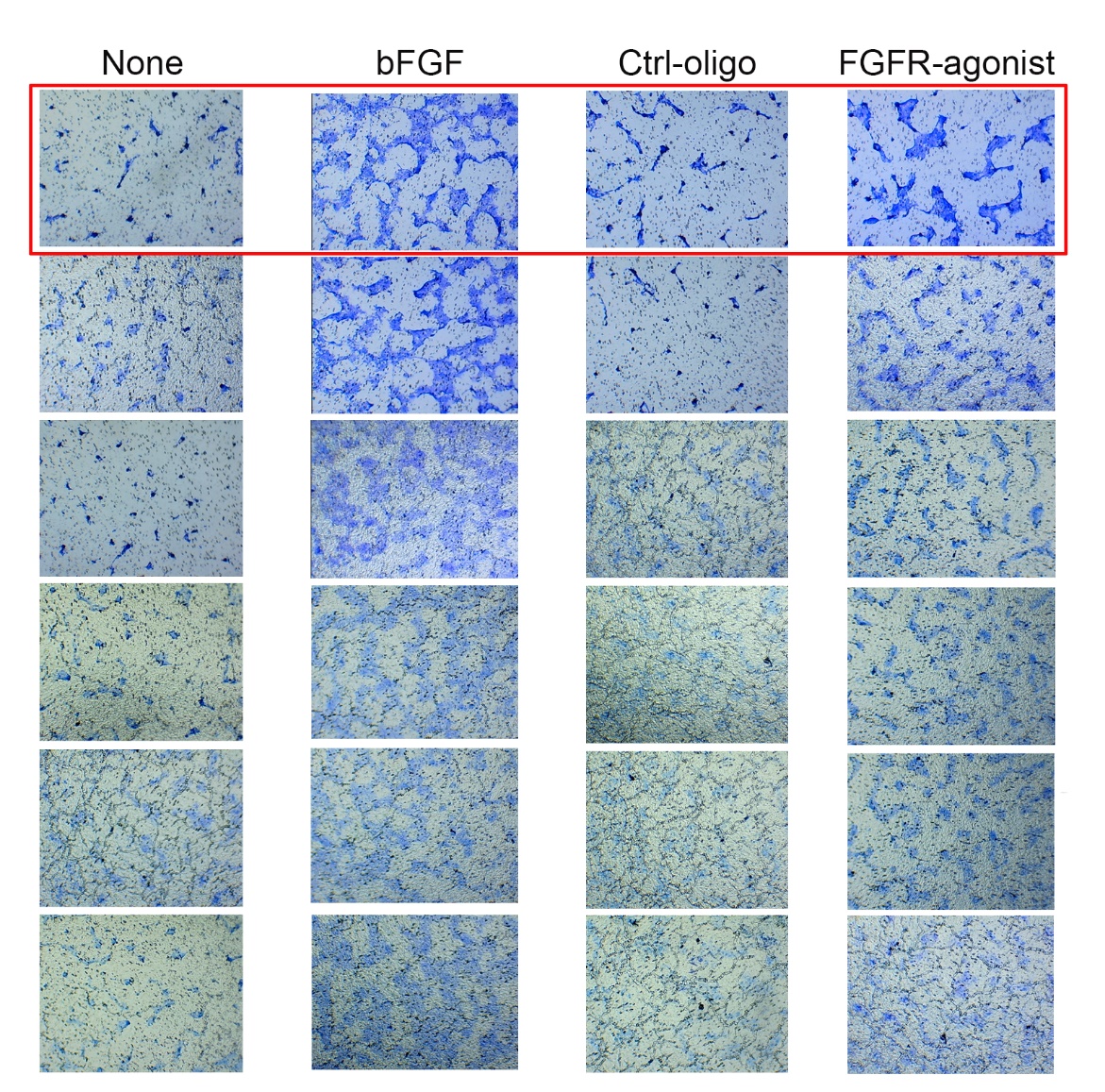


**Fig. S10**. **FGFR-agonist enhances the migratory capability of NPCs.** To assess the directional migration of NPCs, a Transwell assay was conducted. Serum-starved NPCs were placed in the upper chamber, while bFGF(20 ng/mL) and FGFR-agonist (40 nM) were included in the lower chamber to act as chemoattractants. Migration rates were quantified and revealed a substantial increase in the number of NPCs migrating towards FGFR-agonist compared to the Ctrl-oligo treated control group. The experiment was executed with six biological replicates to ensure statistical robustness. The data were subsequently analyzed by ImageJ software and are presented in **Fig. 3C**. Areas highlighted by red boxes represent the selected data used to generate the representative image shown in **Fig. 3B**.


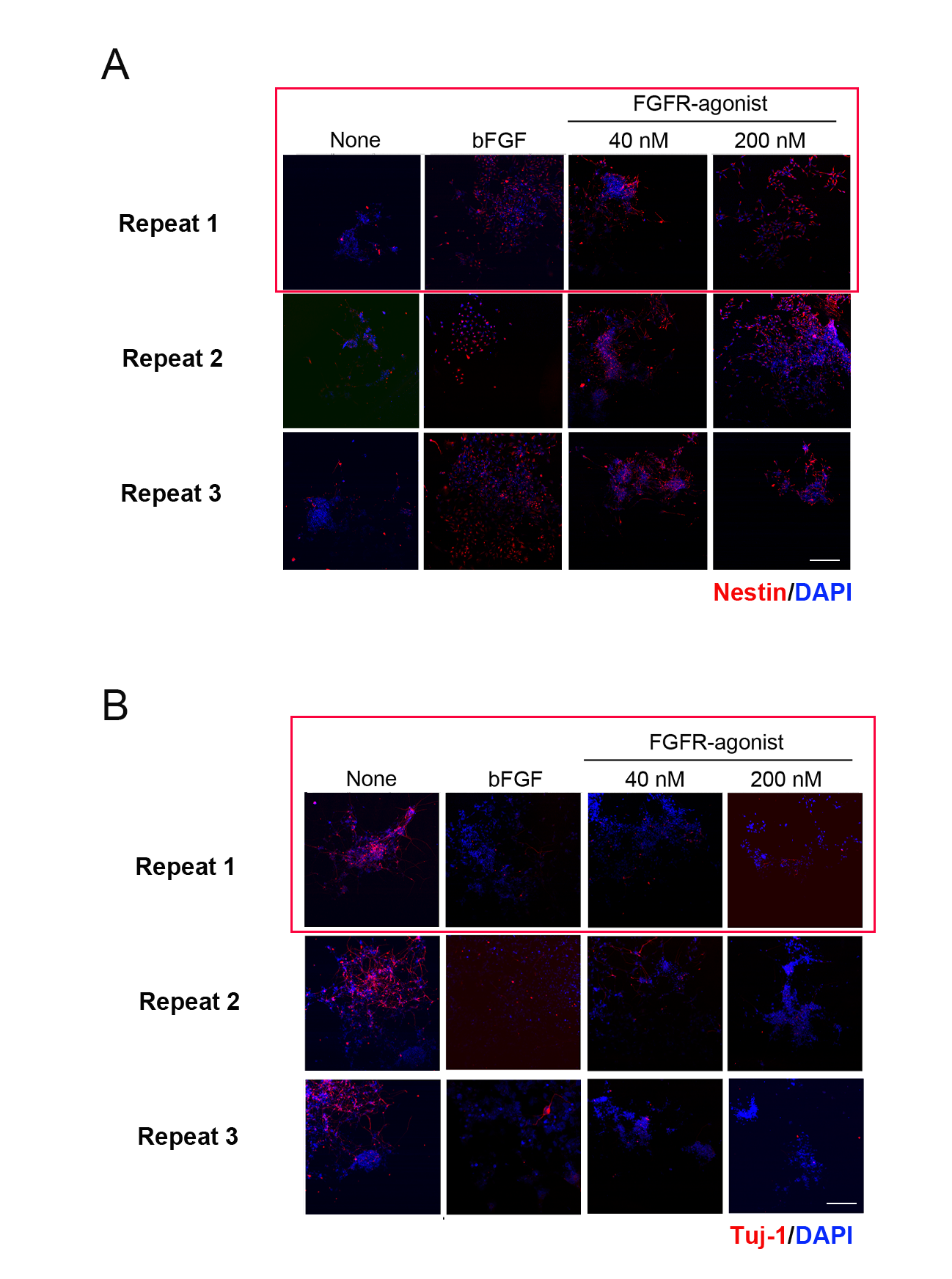


**Fig. S11**. **FGFR-agonist preserves stemness and decouples neuronal differentiation of NPCs.** NPCs were treated with either bFGF or FGFR-agonist at concentrations of 40 nM or 200 nM for 7 days. Immunofluorescence staining was performed to identify and quantify two distinct cell populations, Nestin-positive undifferentiated NPCs (A) and Tuj-1-positive differentiated neurons (B). Each treatment condition was replicated three times to ensure the robustness of the data. Quantitative results from these experiments were compiled for statistical analysis, which is presented in **Fig. 3E**. Areas highlighted by red boxes indicate the data points chosen to generate the representative images in **Fig. 3D**. Scale bar = 100 μm.

**
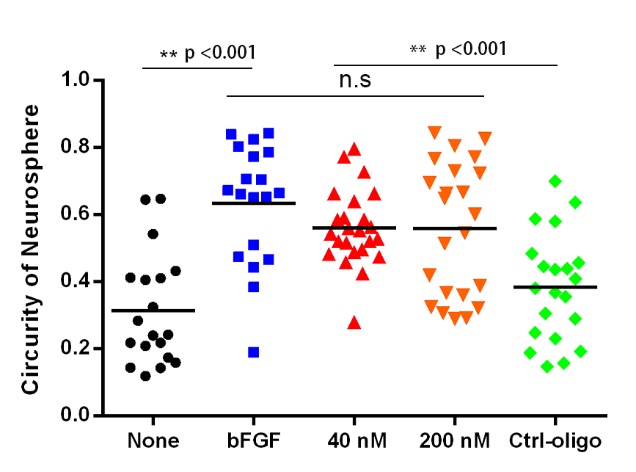
**

**Fig. S12: Stability of neurosphere circularity in response to FGFR-agonist treatment.** NPCs were cultured in a suspension system for 7 days, and two concentrations of FGFR-agonist (40 nM and 200 nM) were tested alongside a standard concentration of bFGF (20 ng/mL). Phase-contrast images were captured using an inverted microscope. For each condition, 15-25 neurospheres were selected, and the circularity was quantified using ImageJ software. Scatter plots were generated based on this analysis. Statistical evaluations were performed using t-tests and one-way ANOVA; **p < 0.001.

**
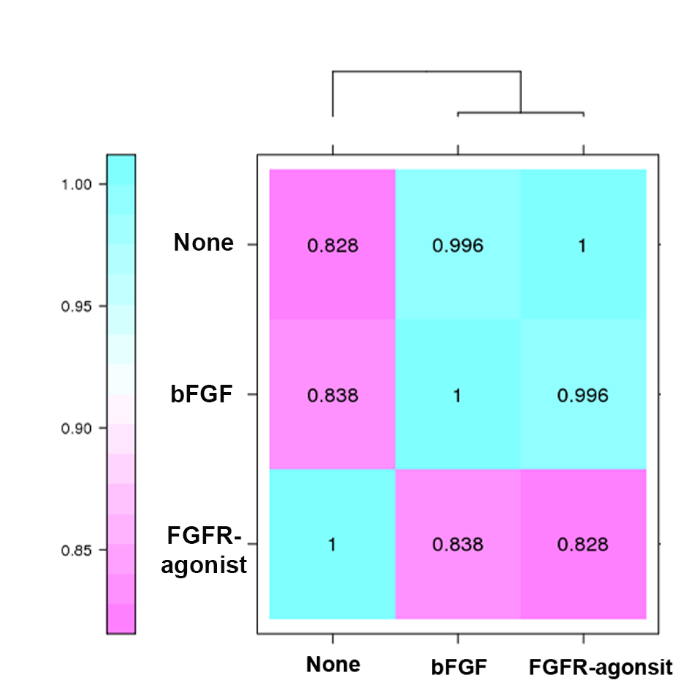
**

**Fig. S13. Correlation heatmap of gene expression levels among the NPCs treated with bFGF and FGFR-Agonist.**


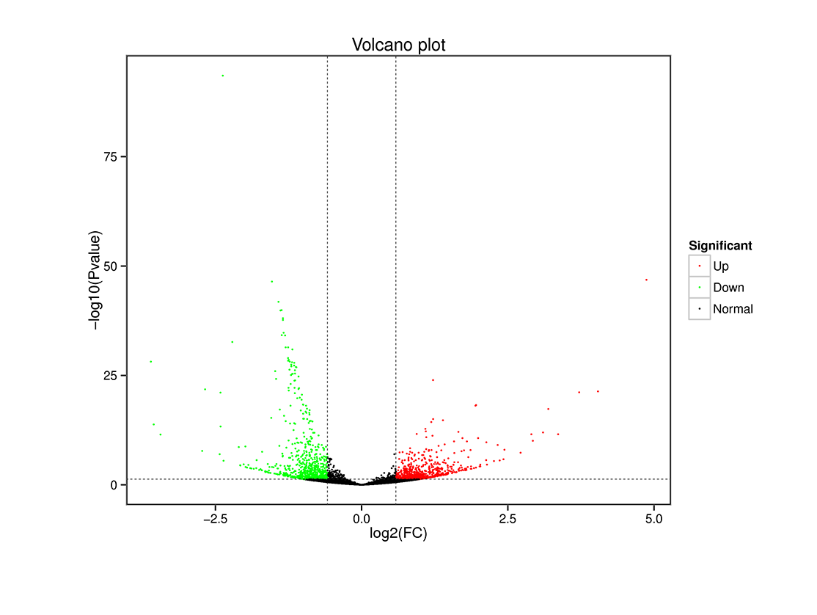


**Fig. S14. Volcano plot illustrating differential gene expression between FGFR-Agonist and Ctrl-oligo treated NPCs groups.** A volcano plot that visually emphasizes the significant differences in transcript expression between NPCs treated with FGFR-agonist and those in the untreated group.

**
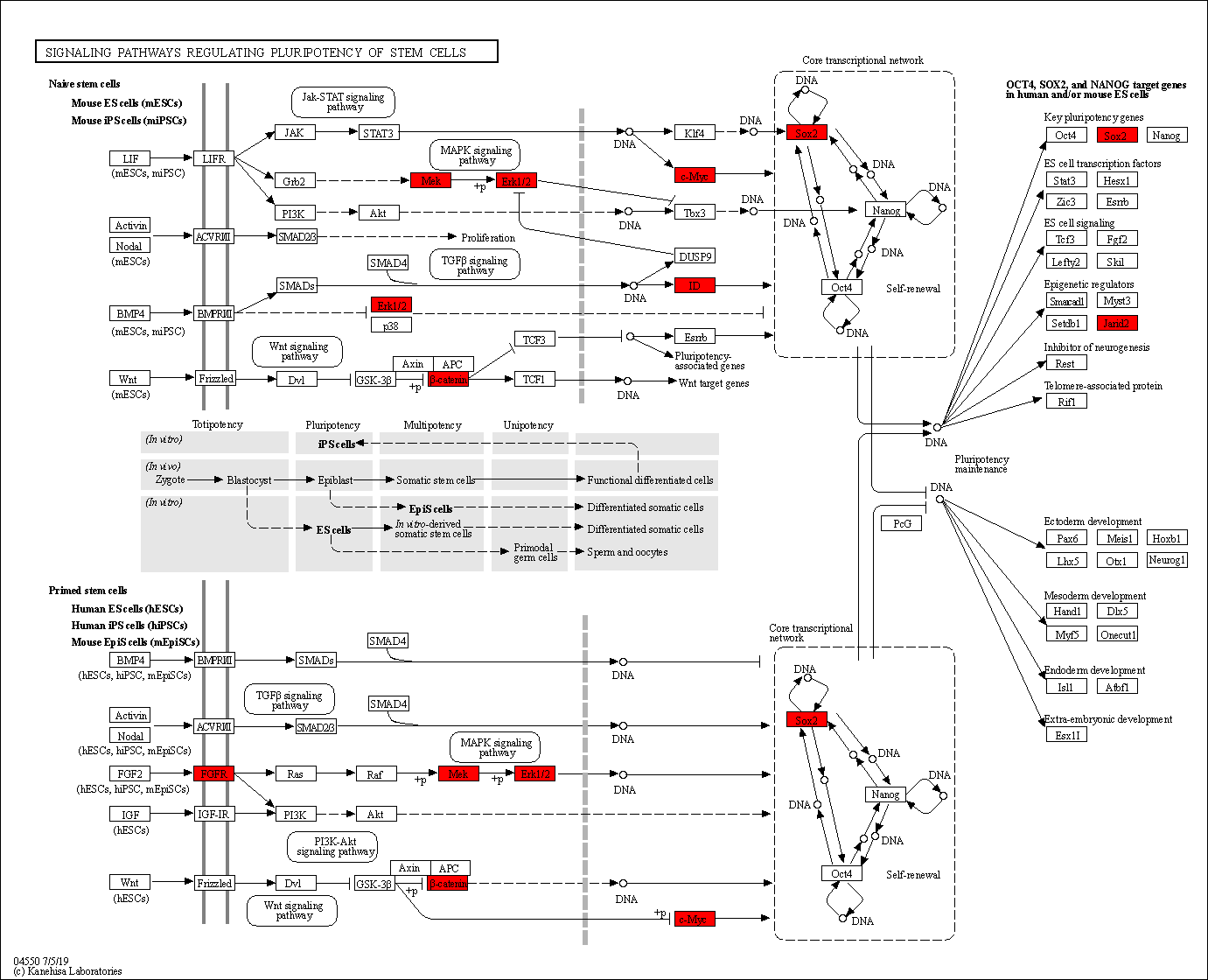
**

**Fig. S15: KEGG pathway analysis illustrating enhanced stemness markers in FGFR-agonist treated NPCs.** The KEGG pathway analysis is employed to pinpoint several key stemness marker genes that display elevated expression following FGFR-agonist treatment. The key upregulated genes, including FGFR, MERK, ERK1/2, Jarid2, SOX2 and c-Myc, were highlighted with red in the KEGG map.
